# International expert panel’s potentially inappropriate prescribing cascades (PIPC) list

**DOI:** 10.1007/s41999-025-01215-x

**Published:** 2025-07-31

**Authors:** Paula A. Rochon, Denis O’Mahony, Antonio Cherubini, Graziano Onder, Mirko Petrovic, Kieran Dalton, Lisa M. McCarthy, Shelley A. Sternberg, Donna R. Zwas, Nathan M. Stall, Christina E. Reppas-Rindlisbacher, Nathalie van der Velde, Sarah N. Hilmer, Wei Wu, Joyce Li, Amy Ly, Jerry H. Gurwitz

**Affiliations:** 1 Women’s Age Lab and Women’s College Research Institute, Women’s College Hospital, 76 Grenville Street, Toronto, ON M5S 1B2 Canada; 2 ICES, Toronto, ON Canada; 3 Division of Geriatric Medicine, Department of Medicine, University of Toronto, Toronto, ON Canada; 4 Institute of Health Policy, Management & Evaluation, Dalla Lana School of Public Health, University of Toronto, Toronto, ON Canada; 5 Sinai Health System, Toronto, ON Canada; 6 Department of Medicine (Geriatrics), School of Medicine, University College Cork, Cork, Ireland; 7 Department of Geriatric Medicine, Cork University Hospital, Wilton, Cork Ireland; 8 Department of Clinical and Molecular Sciences, Università Politecnica Delle Marche, Ancona, Italy; 9 Geriatria, Accettazione geriatrica e Centro di Ricerca per l’invecchiamento, IRCCS INRCA, Ancona, Italy; 10 Department of Geriatrics, Orthopedics and Rheumatology, Università Cattolica del Sacro Cuore, Rome, Italy; 11 Center of Aging, Fondazione Policlinico Universitario Gemelli IRCCS, Rome, Italy; 12 Department of Internal Medicine and Paediatrics, Ghent University, Ghent, Belgium; 13 Pharmaceutical Care Research Group, School of Pharmacy, University College Cork, Cork, Ireland; 14 Leslie Dan Faculty of Pharmacy, University of Toronto, Toronto, ON Canada; 15 Institute for Better Health, Trillium Health Partners, Toronto, ON Canada; 16 Women’s College Research Institute, Women’s College Hospital, Toronto, ON Canada; 17 Department of Medicine, Division of Geriatrics, Maccabi Healthcare Services, Tel Aviv, Israel; 18 Hadassah Medical Center and Faculty of Medicine, Hebrew University of Jerusalem, Jerusalem, Israel; 19 Internal Medicine, Section of Geriatric Medicine, Amsterdam UMC Location University of Amsterdam, Amsterdam, The Netherlands; 20 Amsterdam Public Health Research Institute, Amsterdam, The Netherlands; 21 Department of Clinical Pharmacology, Royal North Shore Hospital, Sydney, Australia; 22 Kolling Institute, Northern Sydney Local Health District and Faculty of Medicine and Health, University of Sydney, Sydney, Australia; 23 Division of Geriatric Medicine, UMass Chan Medical School, Worcester, MA USA

**Keywords:** Prescribing cascade, Delphi consensus, Older adults, Optimizing drug therapy

## Abstract

**Purpose:**

Prescribing cascades contribute to potentially inappropriate prescribing, especially among older adults. With the prescribing cascade framework maturation, it is important to distinguish potentially inappropriate from potentially appropriate prescribing cascades. The objective was to create a comprehensive consensus list of Potentially Inappropriate Prescribing Cascades (PIPCs).

**Methods:**

A prescribing cascade inventory was compiled using published lists. An international panel of 12 experts in geriatric medicine and pharmacology was selected. Panelists participated in a Delphi consensus process completing two questionnaire rounds and one discussion round.

**Results:**

A total of 107 proposed prescribing cascades were identified. After Questionnaire Round 1, 56 prescribing cascades achieved a rating of agree or strongly agree by ≥ 75% of the panelists and were included in the PIPC list. For 32 of the 107 proposed cascades, 50–74% of panelists provided a rating of agree or strongly agree, and were moved to Round 2. In Questionnaire Round 2, 9 of 32 proposed cascades achieved a rating of agree or strongly agree by ≥ 75% of panelists and were included in the final PIPC list. For 14 prescribing cascades, 50–74% of panelists provided a rating of agree or strongly agree, and were included in the Discussion Round. After discussion, no additional prescribing cascades were included.

**Conclusion:**

An explicit list of 65 PIPCs was created using a rigorous Delphi consensus process conducted by international experts on pharmacotherapy for older adults. The PIPC list provides a crucial tool for clinicians and researchers to detect potentially inappropriate prescribing patterns and to foster efforts to improve medication safety.

**Graphical abstract:**

**Supplementary Information:**

The online version contains supplementary material available at 10.1007/s41999-025-01215-x.

## Key summary points

**Aim:**

To create a list of potentially inappropriate prescribing cascades (PIPCs) that should be considered when reviewing a patient’s medications.

**Findings:**

Through a modified Delphi process, an international expert panel reached consensus on 65 potentially inappropriate prescribing cascades to be included in the final PIPC list.

**Message:**

This PIPC list can assist in identifying potentially inappropriate prescribing cascades and help to foster medication safety in older adults.

**Supplementary Information:**

The online version contains supplementary material available at 10.1007/s41999-025-01215-x.

## Graphical abstract

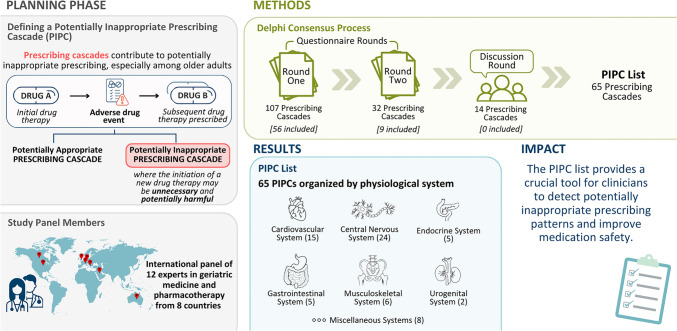

## Introduction

The World Health Organization’s *Medication without Harm* [1] initiative in 2017 emphasizes the need to reduce potentially inappropriate polypharmacy. Prescribing cascades are an important contributor to polypharmacy and potentially inappropriate prescribing, especially among older adults [2]. A prescribing cascade occurs when a drug therapy is initiated (Drug A) and an adverse drug event develops (that is generally mistaken for a new medical condition), leading to a further drug therapy being initiated (Drug B) to manage this new medical condition [3].

The prescribing cascade framework described by Rochon and Gurwitz [3] initially focused on three exemplar prescribing cascades. The number of prescribing cascades currently described in the literature has expanded to more than 160 [4], but there are likely many more. Attention to prescribing cascades has been encouraged in deprescribing protocols [5, 6], emphasized in algorithms designed to optimize prescribing [2, 7], and discussed in medical textbooks and other education resources [8–10]. *ThinkCascades* is a recently developed tool which provides a nine-item list of the most clinically important prescribing cascades affecting older adults [4].

With the maturation of the prescribing cascade framework, it is important to distinguish prescribing cascades that may be potentially inappropriate (where the initiation of a new drug therapy may be unnecessary and potentially harmful) from appropriate prescribing cascades (where the secondary drug therapies are expected, necessary, or beneficial) [11]. This distinction underscores the need for a comprehensive consensus list to be a resource in the clinical care of older adults.

Despite the growing awareness of prescribing cascades, and the many prescribing cascades now identified, the available literature remains limited. Through the efforts described in this paper, we aimed to create a consensus explicit list of what is a potentially inappropriate prescribing cascade (PIPC) using a Delphi consensus process and a panel of international experts.

## Methods

We conducted a modified Delphi process [12] to seek consensus on a comprehensive list of PIPCs.

The Delphi approach was chosen for several reasons. First, given the rapid growth in reports of prescribing cascades, high-quality evidence to support them is often very limited. Consequently, evaluating the existing evidence is not a viable approach for identifying PIPCs. Second, the structured and validated Delphi process allows consensus to be obtained among a panel of international experts, without the biasing effects of direct confrontation or dominant voices [13], increasing the likelihood of a balanced and well-considered outcome.

### Selection and identification of the study conveners and expert Delphi panel members

The study conveners (PR and JG) were members of the Identifying Key Prescribing Cascades in Older People (iKASCADE), an international initiative on drug safety established in 2019 [14].

Twelve panelists, selected based on their expertise in geriatric medicine and clinical pharmacology, were contacted by the study conveners. Eight clinicians from the iKASCADE group were included as panelists, given their unique expertise on prescribing cascades and the international composition of the group (Supplement eTable 1). To ensure gender diversity and to broaden international representation, four additional experts, based on their expertise in geriatric pharmacotherapy, were nominated by iKASCADE group members. All contacted individuals accepted the invitation to be a Delphi panelist and disclosed potential conflicts of interest before engagement.

### Defining a PIPC

**Fig. 1:**
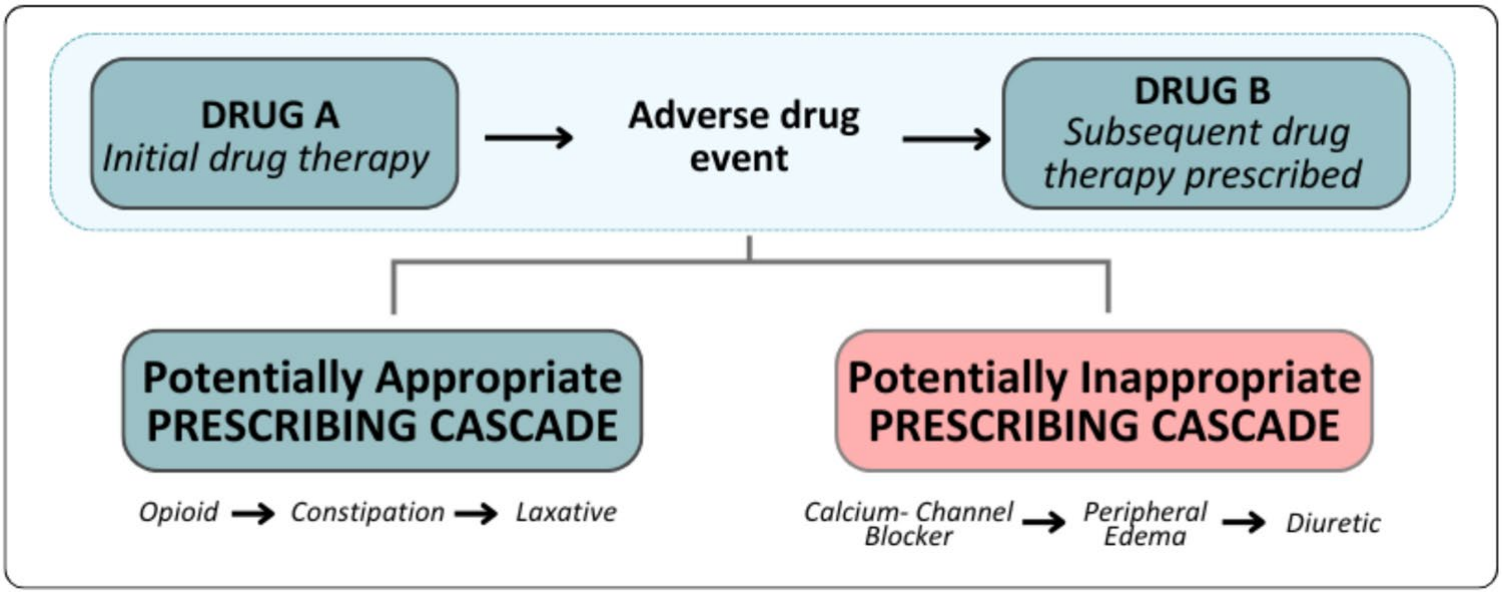
Potentially appropriate and potentially inappropriate prescribing cascade

We leveraged the expertise of the members of the iKASCADE group to seek consensus on the definition of a PIPC. Members of the iKASCADE group, clinicians who are international experts in geriatric medicine and pharmacotherapy for older adults, met to define a PIPC. They specified an example of a PIPC and an example of a potentially appropriate prescribing cascade to help guide the Delphi reviewer’s assessment (see Fig. 1 and Supplement eTable 2).

The iKASCADE group developed a series of options to guide panelists in determining whether a prescribing cascade was potentially inappropriate as outlined below.

For this Delphi questionnaire, when assessing a prescribing cascade, panel members were instructed to assume the following:

1. Drug A was prescribed appropriately for a given clinical indication.
2. Drug A has caused or contributed to the development of the adverse drug event.
3. Drug B was prescribed to treat/manage the adverse drug event caused by Drug A.

When considering whether a prescribing cascade was **potentially inappropriate**, panel members were asked to consider whether it was appropriate or not to have started Drug B. To do this, panel members were instructed to consider the following:

1. The clinical need to prescribe Drug B long term to treat the adverse drug event.
2. Could the prescriber have misinterpreted the adverse drug event as a new symptom unrelated to Drug A, or inappropriately attributed the adverse drug event to the aging process?
3. Whether there are possible alternative strategies to address the adverse drug event (e.g., non-pharmacological alternatives to Drug B, or dose reduction or discontinuation of Drug A, or the use of alternatives to Drug A that are less likely to cause the adverse drug event).

#### Compiling an inventory of prescribing cascades

**Fig. 2:**
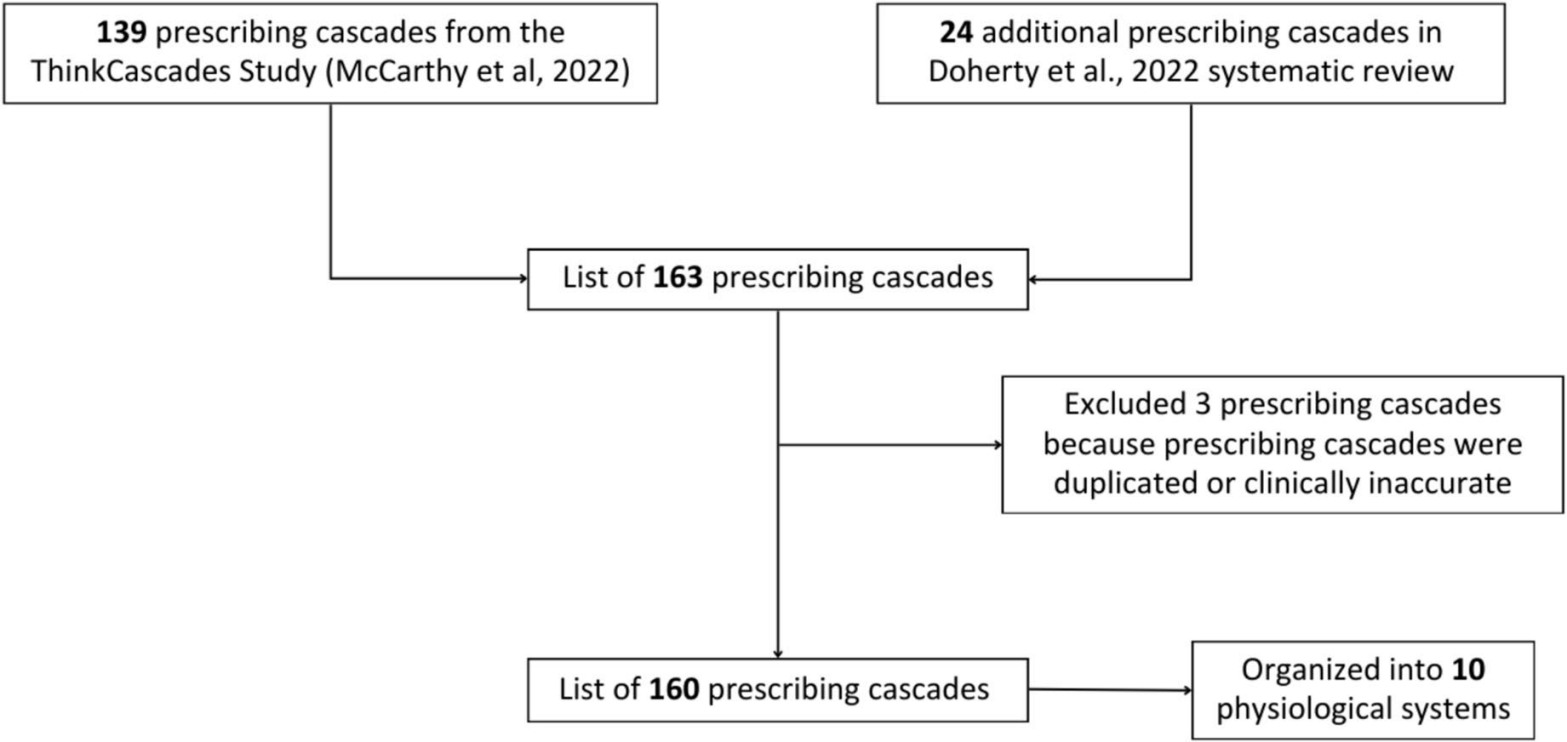
Compiling an inventory of prescribing cascades for evaluation. Legend: The Figure describes the steps followed to compile the inventory of prescribing potentially inappropriate cascades for evaluation

An inventory of prescribing cascades for evaluation in the Delphi questionnaires was compiled using a three-step process (Fig. 2). First, a master list of all previously described prescribing cascades was taken from the inventory of 139 prescribing cascades used in the 2022 ThinkCascades study [4]. An additional 24 prescribing cascades were added from the 2022 systematic review of prescribing cascades led by Doherty et al. [15], yielding 163 prescribing cascades. Two of these prescribing cascades were duplicates and one was incorrectly classified, leaving 160 prescribing cascades for inclusion in our inventory.

Second, the prescribing cascade inventory was organized according to ten physiological systems to align with the STOPP/START criteria. Within each system, the inventory was further organized alphabetically based on Drug A, the drug therapy initiating the prescribing cascade. Finally, for each prescribing cascade, selected references relating to the prescribing cascade were provided, with the literature describing these many prescribing cascades being highly variable in study quality.

#### Modified Delphi study design

**Fig. 3:**
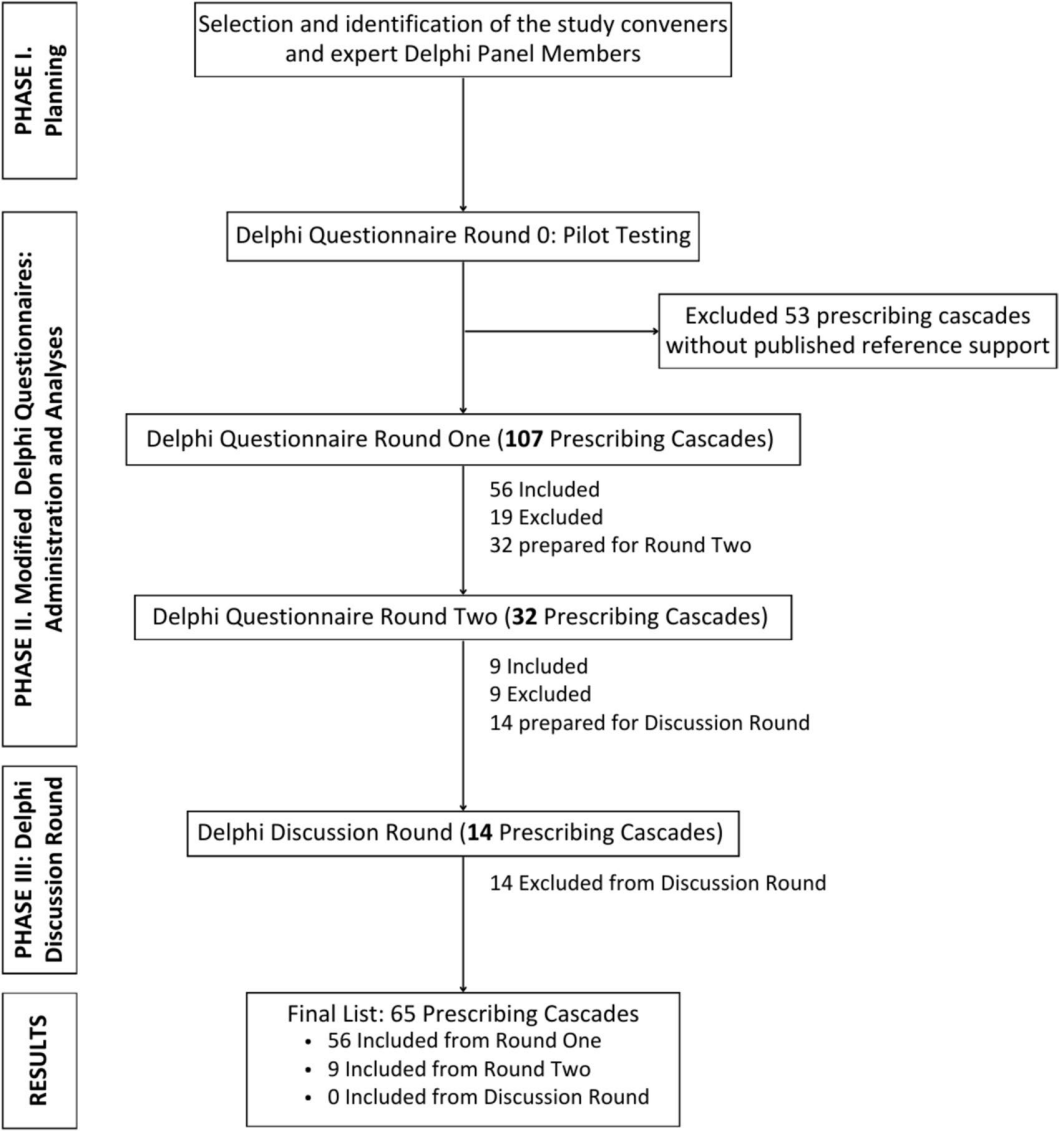
Delphi process to seek consensus on an extensive list of Potentially Inappropriate Prescribing Cascades (PIPC). Legend: The Figure describes the three phases followed to create the consensus list of 65 Potentially Inappropriate Prescribing Cascades (PIPCs)

We followed a modified Delphi method that follows a structured and validated process to achieve consensus on our comprehensive list of potentially inappropriate prescribing cascades. The three phases were: Planning, Delphi Questionnaires (Round 0, 1, and 2), and Discussion Round—as summarized in Fig. 3. The Delphi Questionnaire and Discussion Round were administered between June and September 2024. The online survey platform REDCap [16] captured responses to the questionnaires. Study data were collected and managed using REDCap electronic data capture tools hosted at Women’s College Hospital [17, 18] (See Supplement eAppendix 1).

Our questionnaire was based on the ThinkCascades study methodology [4] and aligned with the consensus approach employed in similar initiatives relating to developing resources to guide prescribing for older adults [19]. Throughout, we adhered to RAND Methodological Guidance for Conducting and Critically Appraising Delphi Panels [12], and the Conducting and Reporting Delphi Studies (CREDES) Checklist [20] (Supplement eTable 3) to ensure a systematic and consensus-driven approach.

The study was approved by the Research Ethics Board of Women’s College Hospital (REB #: 2024-0011-E). Informed consent was obtained from panel members. The protocol was registered in Open Science Framework (https://doi.org/10.17605/OSF.IO/7DF62).

#### Phase I: planning

In the planning phase, we created the inventory of prescribing cascades, and identified the published literature related to each prescribing cascade.

Panel members’ demographic information including their gender, country of origin, and clinical area of expertise was collected (Supplement eTable 1). Partial panelist anonymity was provided; specifically, while panelists were known to each other, their individual prescribing cascades ratings during the Delphi process were not shared.

#### Phase II: modified Delphi questionnaires—administration and analyses.

Our Delphi methodology consisted of Round 0 (pilot testing) and two Delphi Questionnaire Rounds (Round 1 and 2). Panel members were provided with aggregate data following each Delphi round but did not know how individual panel members responded to any part of the questionnaires.

##### Questionnaire Round 0: pilot testing

The Delphi Questionnaire was pilot tested to identify and address issues that might hinder panel member retention or their ability to respond to questions. This testing also provided an opportunity to explore logistical issues with the REDCap program [16], address any confusion or uncertainty around responses, and optimize the value of references.

The study conveners (PR and JHG) and two iKASCADE group members (DO’M and AC) conducted the pilot testing. Any problems with the online questionnaire platform were identified and addressed. First, the survey was long with 160 proposed prescribing cascades for review including 53 prescribing cascades with no references. Given that prescribing cascades without references were less well established, the list was restricted to those with at least one published reference, reducing the inventory to 107 prescribing cascades (see Supplemental eTable 4 for all prescribing cascades and their respective references). The second issue arose from the questionnaire’s complexity and the use of REDCap features unfamiliar to some panel members. To address this issue, a 30-min group Delphi panel member video conference orientation session was provided, to explain the Delphi panel goals and provide guidance on REDCap platform logistics. Panel members unable to join the group session were provided with a personal session.

##### Questionnaire Round 1

Panel members were given a 2-week period to complete the online questionnaire. The first section of the questionnaire provided general study information, explained the study's objectives, procedures, risks, and benefits, and then asked panel members to submit their electronic consent to participate. A total of 107 prescribing cascades were presented to panel members for evaluation. References specific to each prescribing cascade were available using a digital link.

Each panel member rated the degree to which they agreed that each prescribing cascade was potentially inappropriate using a 5-point Likert scale (Strongly agree, Agree, Neutral, Disagree, Strongly disagree). Next, panel members were asked ‘which single factor was the most influential in your rating’ to better understand their thought process in decision-making. If they had chosen ‘Strongly agree’ or ‘Agree’ on the 5-point Likert scale, they were provided the following options:

- Drug B is potentially inappropriate.
- Availability of possible alternative strategies to Drug B to manage the adverse drug event (e.g., reducing the dose of Drug A or switching to an alternative safer non-pharmacologic or pharmacologic treatment).
- Consideration of the clinical need to maintain the patient on Drug A despite the occurrence of the adverse drug event.
- The severity of the adverse drug event.
- The likelihood of the adverse drug event being misinterpreted as a new medical condition unrelated to medication exposure or merely attributed to the aging process.
- Other reason.

If they chose ‘Neutral’, ‘Disagree’, or ‘Strongly disagree’ on the 5-point Likert scale, they had the following options:

- This is an appropriate approach to care.
- I am not convinced that this is a potentially inappropriate prescribing cascade.
- Other reason.

Panel members were also allowed to provide free text comments for each proposed prescribing cascade. These comments were subsequently provided to the panel members for their consideration during the Discussion round.

The rules for inclusion of a potential prescribing cascades on our comprehensive PIPC list was that at least 75% of panel members strongly agreed or agreed. Potential prescribing cascades for which less than 50% of panel members strongly agreed or agreed were excluded from further consideration. This threshold was chosen to be consistent with the approach used in the development of STOPP/START potentially inappropriate prescribing criteria [19].

##### Questionnaire Round 2

In Questionnaire Round 2, where consensus could not be reached to include or exclude particular prescribing cascades (i.e., between 50 and 74% of panelists selected strongly agreed or agreed) in Questionnaire Round 1, these proposed cascades were re-evaluated using the same questions. In this round, panel members each received a customized report showing their rating for each proposed prescribing cascade and a histogram summarizing the ratings of all panel members. Prescribing cascades for which consensus could not be reached moved on to the Discussion Round.

#### Phase III: Discussion Round

Panel members met online for a final Discussion Round moderated by the study conveners (PR and JHG). Given the international nature of our Delphi panel, spanning multiple time zones, three Discussion Round meetings were organized to capture the input of all panelists. At the Discussion Round, a multi-step process was used. First, a histogram for each prescribing cascade was presented showing the group ratings of the prescribing cascade. Second, the contents of the references linked to the prescribing cascades were described. Panel members were then asked to discuss all the presented material. Finally, using an anonymous polling tool on the online video conference platform, each panel member again rated the extent to which they agreed an example was a PIPC. Prescribing cascades were included in the final comprehensive list if at least 75% panel members strongly agreed or agreed.

## Results

Panel members included six male and six female experts (physicians, pharmacologists, and pharmacists) from eight countries (i.e., Australia, Belgium, Canada, Ireland, Israel, Italy, The Netherlands, and United States), as summarized in Supplement eTable 1. There was 100% participation of the panel members in the two Questionnaire Rounds and the Discussion Round.

### Questionnaire Round 1

As depicted in Fig. 3 and summarized in Supplement eTable 5, 56 of 107 prescribing cascades from the extensive list achieved a rating of agree or strongly agree by ≥ 75% of the panelists in Questionnaire Round 1, and therefore were included in the final PIPC list. For 32 of 107 prescribing cascades, 50–74% of panelists provided a rating of ‘agree’ or ‘strongly agree’ and were included in the Questionnaire Round 2. For 19 of 107 prescribing cascades, less than 50% of panelists provided a rating of ‘agree’ or ‘strongly agree’ and were excluded from the final comprehensive list.

When asked about factors considered when rating prescribing cascades, Delphi panel members provided the following information. For prescribing cascades considered to be potentially inappropriate, the most influential factors in rating were: ‘Availability of possible alternative strategies to Drug B to manage the adverse drug event’ (54%) and ‘The likelihood of the adverse drug event being misinterpreted as a new medical condition’ (31%). Where respondents provided a ‘disagree’, ‘strongly disagree’, or ‘neutral’ response on whether each cascade was a PIPC, the most influential factors in rating were: ‘I am not convinced that this is a potentially inappropriate prescribing cascade’ (44%) and ‘This is an appropriate approach to care’ (42%).

### Questionnaire Round 2

There were 32 proposed prescribing cascades evaluated in the Questionnaire Round 2. As depicted in Fig. 3 and summarized in Supplement eTable 6, 9 of 32 prescribing cascades from the inventory achieved a rating of ‘agree’ or ‘strongly agree’ by ≥ 75% of the panelists and were included in the final PIPC list. Nine of 32 prescribing cascades with less than 50% of panelists indicating ‘agree’ or ‘strongly agree’ were excluded. For 14 of the 32 proposed prescribing cascades, 50–74% of panelists provided a rating of ‘agree’ or ‘strongly agree’, and they were included in the Discussion Round.

### Discussion Round

After the Discussion Round, no proposed prescribing cascades met the inclusion criteria, and all remaining 14 proposed PIPCs were excluded from the PIPC list (Supplement eTable 7).

### Final list of PIPCs

Through this Delphi process, a comprehensive list of 65 PIPCs was created (see Table 1). This list was then reviewed by the study conveners and iKASCADE panel members. During this process, three refinements were made; the details of these refinements and the final agreed list of PIPCs are illustrated in Supplement eAppendix 2. The PIPC list has been endorsed by the European Geriatric Medicine Society (EuGMS) with representatives from more than 60 countries.

**Table 1:** Comprehensive PIPC list of 65 potentially inappropriate prescribing cascades arranged by physiological system

Drug A	Adverse event	Drug B
**Cardiovascular system**		
*Drug Class*		
Angiotensin converting enzyme inhibitor	Cough	Cough remedy
Antihypertensive	Orthostatic hypotension/dizziness	Antiemetic
Beta-blocker (particularly lipophilic e.g., propranolol)	Depression	Antidepressant
Beta-blocker (particularly lipophilic e.g., propranolol)	Erectile dysfunction	Phosphodiesterase-5 inhibitor, alprostadil
Calcium channel blocker	Peripheral edema	Diuretic
Calcium channel blocker	Constipation	Laxative
Diuretic	Gout or Hyperuricemia	Anti-gout agent
Diuretic	Urinary incontinence	Overactive bladder medication
Hydroxymethylglutaryl-coenzyme A (HMG Co-A) reductase inhibitor (statin)	Myalgia/myositis	
		Pain reliever
HMG Co-A reductase inhibitor	Myalgia/myositis	Mineral supplement
HMG Co-A reductase inhibitor	Myalgia/myositis	Quinine sulfate
HMG Co-A reductase inhibitor	Insomnia	Sleep agent
*Individual Drug*		
Digoxin	Nausea	Antiemetic
Midodrine	Hypertension	Antihypertensive
**Central nervous system**		
*Drug Class*		
Anticonvulsant	Rash	Topical corticosteroid
Anticonvulsant	Nausea	Antiemetic
Antipsychotic	Extrapyramidal symptoms	Beta-blocker
Antipsychotic	Extrapyramidal symptoms	Antiparkinsonian agent
Antipsychotic	Extrapyramidal symptoms	Anti-tremor antimuscarinic
Antipsychotic	Akathisia or tardive movements	Sedative
Antipsychotic	Arrhythmia	Antiarrhythmic
Antipsychotic	Hyperglycemia	Antihyperglycemic
Benzodiazepine	Cognitive impairment	Cholinesterase Inhibitor
Cholinesterase inhibitor	Urinary incontinence	Overactive bladder medication
Cholinesterase inhibitor	Insomnia	Sleep agent
Cholinesterase inhibitor	Gastrointestinal upset	Antiemetic
Cholinesterase inhibitor	Gastrointestinal upset	Bismuth subsalicylate
Cholinesterase inhibitor	Diarrhea	Antidiarrheal
Cholinesterase inhibitor	Rhinorrhea	Antihistamine
Dopaminergic Antiparkinsonian agent	Psychotic symptoms, hallucinations	Antipsychotic
Gabapentinoid	Peripheral edema	Diuretic
Selective serotonin reuptake inhibitor/Serotonin and norepinephrine reuptake inhibitor (SSRI/SNRI)	Urinary Incontinence	Overactive bladder medication
Tricyclic antidepressant	Cognitive Impairment	Cholinesterase Inhibitor
Tricyclic antidepressant	Constipation	Laxative
Tricyclic antidepressant	Urinary incontinence	Overactive bladder medication
*Individual Drug*		
Flunarizine	Depression	Antidepressant
Lithium	Extrapyramidal symptoms	Antiparkinsonian agent
Venlafaxine	Hypertension (dose-related)	Antihypertensive
Venlafaxine	Tremor	Benzodiazepine
**Endocrine system**		
*Drug Class*		
Dipeptidyl peptidase 4 (DPP-4) inhibitor (e.g., sitagliptin, saxagliptin)	Joint pain	Nonsteroidal anti-inflammatory drug
Sodium-glucose cotransporter-2 (SGLT-2) inhibitor	Mycotic genital infections	Antifungal
*Individual Drug*		
Metformin	Diarrhea	Antidiarrheal
Pioglitazone or Rosiglitazone	Edema	Diuretic
Rosiglitazone	Heart failure	Diuretic
**Gastrointestinal system**		
*Drug Class*		
Anticholinergic antiemetic	Urinary retention	Alpha-1 receptor blocker
Antidopaminergic antiemetic	Extrapyramidal symptoms	Antiparkinsonian agent
Laxative	Diarrhea	Antidiarrheal agent
Proton pump inhibitor	Osteoporosis, fractures	Vitamin supplement
Proton pump inhibitor	Vitamin or Mineral Deficiency	Vitamin or Mineral Deficiency
**Musculoskeletal system**		
*Drug Class*		
Bisphosphonate	Gastritis	Gastroprotective agent
Nonsteroidal anti-inflammatory drug (NSAID)	Gastritis/gastric ulcer/gastrointestinal bleed	Gastroprotective agent
NSAID	Nausea	Antiemetic
NSAID	Hypertension	Antihypertensive
NSAID	Worsening of heart failure	Heart failure agent
Opioid	Depression	Antidepressant
**Urogenital system**		
*Drug Class*		
Alpha-1 receptor blocker	Orthostatic hypotension, dizziness	Vestibular suppressant
Urinary anticholinergic	Dry mouth	Saliva substitute
**Miscellaneous system**		
*Drug Class*		
Carbapenem (e.g., imipenem, meropenem, ertapenem)	Seizures	Anticonvulsant
Corticosteroid	Insomnia	Sleep agent
Corticosteroid	Psychosis	Antipsychotic
Corticosteroid	Hypertension	Antihypertensive
*Individual Drug*		
Acitretin	Vulvo-vaginal candidiasis	Antifungal
Erythromycin	Arrhythmia	Antiarrhythmic
Fludrocortisone	Hypertension	Antihypertensive
Iron supplement	Constipation	Laxative

The potentially inappropriate prescribing cascades (PIPCs) were organized according to the physiological systems to align with the STOPP/START criteria. Within each system, the PIPCs were organized by drug classes followed by individual drugs and listed alphabetically based on Drug A, the drug therapy initiating the prescribing cascade

## Discussion

Using a modified Delphi consensus approach involving experts in drug prescribing for older adults from eight countries, we created an International Expert Panel’s Comprehensive List of Potentially Inappropriate Prescribing Cascades (PIPCs). Our comprehensive PIPC list comprises 65 potentially inappropriate prescribing cascades, created using a rigorous Delphi consensus process. Including experts in pharmacotherapy in older people from eight countries is a strength of this study and ensures the broad applicability of this list in clinical practice.

Our comprehensive PIPC list adds to the array of pharmacological risk assessment tools that are designed to optimize prescribing for older adults, including STOPP/START criteria [19], Beers criteria [21], EURO-FORTA criteria [22], and the Drug Burden Index [23]. Like these existing risk assessment tools, we anticipate that the PIPC list will need to be updated on a regular basis. This list can be integrated into these existing risk assessment tools, clinical workflows such as electronic health records, and inform policy changes in geriatric pharmacology. The alignment of the PIPC list with the STOPP/START physiological systems will facilitate its integration.

Our explicit list of 65 PIPCs provides another set of explicit prescribing criteria for clinicians and healthcare organizations to employ during routine medication review in multimorbid older people exposed to varying degrees of polypharmacy. Identifying prescribing cascades on the PIPC list complements the existing tools to optimize prescribing for older adults. The adoption of this list will need to consider potential barriers, such as clinician resistance to deprescribing, lack of awareness about prescribing cascades, and time constraints. Our hope is that by having a list of potentially inappropriate prescribing cascades to use during routine medication review, along with complementary education and clinical decision support tools, we will address these barriers and contribute to reducing potentially inappropriate prescribing and problematic polypharmacy.

Because all long-term medications (i.e. prescribed for more than 2 weeks) should be reviewed on a regular basis, our PIPC list could be included as part of a comprehensive medication review process. The PIPC list adds value to these medication reviews as it facilitates the identification of potentially inappropriate drug combinations. The presence of a PIPC highlights that the patient is treated with a potentially inappropriate drug (or drugs), suggesting that their overall pharmacological therapy should be carefully reviewed. Integrating the PIPC list into clinical decision support tools could further aid prescribers in real-time medication reviews, and minimize potentially inappropriate prescribing at the point of care.

Prescribing cascades on the PIPC list can complement the existing clinical tools designed to optimize prescribing and can facilitate the identification of inappropriate drug combinations during routine review of medications. The list focuses on PIPCs rather than assuming that all prescribing cascades are problematic and emphasizes that once identified, PIPCs require further review with the prescriber and the patient to determine whether these medications are the most appropriate combination for the individual patient’s circumstances at that particular time. Moreover, the presence of a PIPC might highlight that the patient is treated with a potentially inappropriate drug (or drugs), suggesting that the overall pharmacological therapy of the patients should be carefully reviewed. Integrating the PIPC list into clinical decision support tools can aid prescribers in real-time medication reviews, minimizing inappropriate prescribing at the point of care.

The impact of prescribing cascades on patient outcomes remains understudied. Existing prescribing cascade research has used administrative data to explore adverse events associated with prescribing cascades, leading to emergency room visits and hospitalizations [11]. In the future, there is an opportunity for clinical research to explore the benefit of addressing drug combinations on the PIPC list for reducing adverse drug events and improving medication safety. For example, evaluating the benefit of integrating the PIPC list into clinical decision support tools to minimize inappropriate prescribing at the point of care.

Measuring the population-level impact of prescribing cascades from the PIPC list is a next step to inform targeted interventions. For example, prescription sequence symmetry analysis (PSSA) that measures the association between two medications [24, 25] can be combined with population prevalence data for the filling of prescriptions for the drug therapies on the PIPC list. Taken together, these data can be used to prioritize the prescribing cascades that have high strengths of association, and are common, among those with specific physiological system conditions for more targeted population-level interventions. As such, the PIPC list can be deployed to assess potentially inappropriate prescribing cascade prevalence in older adults with specific physiological system conditions. These types of analyses can be performed using health administration data [26–28]. Depending on the potential prescribing cascade prevalence, the PIPC list could conceivably be used as a prescribing cascade prevention tool in future intervention studies.

### Limitations

We acknowledge a number of limitations with this project. First, the quality of the published literature describing prescribing cascades was highly variable. Most prescribing cascades evaluated through this Delphi consensus process were identified from case reports, small scale case series, and review articles. Others were extrapolated from PSSA [24, 25], and some from rigorously conducted retrospective cohort studies exploring prescribing cascades at a population level [26, 28]. The variability in the quality of published evidence, and the recognition that prescribing cascades can in some cases be potentially appropriate and in others potentially inappropriate, made it important to use expert opinion through the Delphi consensus process. Next, we acknowledge that our PIPC list will not be complete given that the number of prescribing cascades is theoretically vast, [29] and growing as new evidence emerges. Nevertheless, we contend that our explicit PIPC list should be a starting point in more common PIPC detection and should encourage clinicians to consider additional PIPCs not included in the present PIPC list.

Our Delphi process considered prescribing cascades identified from recently published lists, which were also supported by references. Our list does not include PIPCs that have not yet been reported in the literature. Further, while our panel included experts from eight countries, regional variation in the use of drug therapies and tailored approaches will still need to be considered when implementing the PIPC list.

### Implications

Our systems-based explicit list brings PIPCs to the attention of clinicians. Prescribing cascades may represent potentially inappropriate polypharmacy and, in some cases, associated with serious adverse events leading to emergency room visits and hospitalizations [11] that are costly to patients and the healthcare system. Our list identifies 65 clinically relevant PIPCs, is organized by physiological system, and lists Drug A in alphabetical order, all to facilitate ease of use in clinical practice. As more PIPCs are identified, this list will require updates. Creating a digital version of this list, or incorporating this list into electronic health records, could facilitate its use as part of the medication review process in clinical practice.

## Conclusions

We present a physiological systems-based explicit list of 65 Potentially Inappropriate Prescribing Cascades (PIPCs) using a rigorous Delphi consensus process conducted by international experts from eight countries on drug prescribing for older adults. This PIPC list brings attention to an important and underrecognized aspect of suboptimal or unsafe prescribing in older people. The PIPC list provides a crucial tool for clinicians and researchers to detect potentially inappropriate prescribing patterns and to foster efforts to improve medication safety.

## Supplementary Information

Below is the link to the electronic supplementary material.

Supplementary file 1 (DOCX 929 KB)

## Data Availability

The sponsor was not involved in the design, methods, subject recruitment, data collection, analysis, or preparation of the manuscript.
