# Correction: International expert panel’s potentially inappropriate prescribing cascades (PIPC) list

**DOI:** 10.1007/s41999-025-01378-7

**Published:** 2025-12-18

**Authors:** Paula A. Rochon, Denis O’Mahony, Antonio Cherubini, Graziano Onder, Mirko Petrovic, Kieran Dalton, Lisa M. McCarthy, Shelley A. Sternberg, Donna R. Zwas, Nathan M. Stall, Christina E. Reppas-Rindlisbacher, Nathalie van der Velde, Sarah N. Hilmer, Wei Wu, Joyce Li, Amy Ly, Jerry H. Gurwitz

**Affiliations:** 1 Women’s Age Lab and Women’s College Research Institute, Women’s College Hospital, 76 Grenville Street, Toronto, ON M5S 1B2 Canada; 2 ICES, Toronto, ON Canada; 3 Division of Geriatric Medicine, Department of Medicine, University of Toronto, Toronto, ON Canada; 4 Institute of Health Policy, Management & Evaluation, Dalla Lana School of Public Health, University of Toronto, Toronto, ON Canada; 5 Sinai Health System, Toronto, ON Canada; 6 Department of Medicine (Geriatrics), School of Medicine, University College Cork, Cork, Ireland; 7 Department of Geriatric Medicine, Cork University Hospital, Wilton, Cork Ireland; 8 Department of Clinical and Molecular Sciences, Università Politecnica Delle Marche, Ancona, Italy; 9 Geriatria, Accettazione Geriatrica e Centro Di Ricerca Per L’invecchiamento, IRCCS INRCA, Ancona, Italy; 10 Department of Geriatrics, Orthopedics and Rheumatology, Università Cattolica del Sacro Cuore, Rome, Italy; 11 Center of Aging, Fondazione Policlinico Universitario Gemelli IRCCS, Rome, Italy; 12 Department of Internal Medicine and Paediatrics, Ghent University, Ghent, Belgium; 13 Pharmaceutical Care Research Group, School of Pharmacy, University College Cork, Cork, Ireland; 14 Leslie Dan Faculty of Pharmacy, University of Toronto, Toronto, ON Canada; 15 Institute for Better Health, Trillium Health Partners, Toronto, ON Canada; 16 Women’s College Research Institute, Women’s College Hospital, Toronto, ON Canada; 17 Department of Medicine, Division of Geriatrics, Maccabi Healthcare Services, Tel Aviv, Israel; 18 Hadassah Medical Center and Faculty of Medicine, Hebrew University of Jerusalem, Jerusalem, Israel; 19 Internal Medicine, Section of Geriatric Medicine, Amsterdam UMC Location University of Amsterdam, Amsterdam, The Netherlands; 20 Amsterdam Public Health Research Institute, Amsterdam, The Netherlands; 21 Department of Clinical Pharmacology, Royal North Shore Hospital, Sydney, Australia; 22 Kolling Institute, Northern Sydney Local Health District and Faculty of Medicine and Health, University of Sydney, Sydney, Australia; 23 Division of Geriatric Medicine, UMass Chan Medical School, Worcester, MA USA

**Correction: European Geriatric Medicine (2025) 16:1573–1584** 10.1007/s41999-025-01215-x

The article “International expert panel’s potentially inappropriate prescribing cascades (PIPC) list”, written by Paula A. Rochon1,2,3,4,5 · Denis O’Mahony6,7 · Antonio Cherubini8,9 · Graziano Onder10,11 · Mirko Petrovic12 · Kieran Dalton13 · Lisa M. McCarthy14,15,16 · Shelley A. Sternberg17 · Donna R. Zwas18 · Nathan M. Stall1,3,5 · Christina E. Reppas Rindlisbacher1,3 · Nathalie van der Velde19,20 · Sarah N. Hilmer21,22 · Wei Wu1 · Joyce Li1 · Amy Ly1 · Jerry H. Gurwitz23, was originally published under exclusive license to © © The Author(s), under exclusive licence to European Geriatric Medicine Society 2025. As a result of the subsequent decision to publish the article under the open access model, the article’s copyright notice was changed on 30 September 2025 to © The Author(s) 2025 and the article is now distributed under a Creative Commons Attribution. To view a copy of this licence, visit http://creativecommons.org/licenses/by/4.0/.

