# Supplementary material for: International expert panel’s potentially inappropriate prescribing cascades (PIPC) list
Source: Eur Geriatr Med. 2025 Jul 31;16(5):1573–84. doi: 10.1007/s41999-025-01215-x (PMC12712104; doi:10.1007/s41999-025-01215-x)
Supplement: Supplementary file 1 — (DOCX 929 KB) [file 41999_2025_1215_MOESM1_ESM.docx]

**International Expert Panel’s *Potentially Inappropriate Prescribing Cascades (PIPC) List***

Paula A. Rochon MD MPH^a-e^, Denis O’Mahony MD DSc^f,g^, Antonio Cherubini MD PhD^h,i^, Graziano Onder MD^j,k^, Mirko Petrovic MD PhD^l^, Kieran Dalton PhD^m^, Lisa M. McCarthy PharmD MSc^n-p^, Shelley A. Sternberg MD^q^, Donna R. Zwas MD MPH^r^, Nathan M. Stall MD PhD^a,c,e^, Christina E. Reppas-Rindlisbacher MD^a,c^, Nathalie van der Velde PhD^s,t^, Sarah N Hilmer MBBS PhD^u,v^, Wei Wu MSc^a^, Joyce Li MSc^a^, Amy Ly MSc^a^, Jerry H. Gurwitz MD^w^

^a^ Women’s Age Lab and Women’s College Research Institute, Women’s College Hospital, Toronto, Ontario, Canada

^b^ ICES, Toronto, Ontario, Canada

^c^ Department of Medicine, University of Toronto, Toronto, Ontario, Canada

^d^ Institute of Health Policy, Management & Evaluation, Dalla Lana School of Public Health, University of Toronto, Toronto, Ontario, Canada

^e^ Sinai Health System, Toronto, Ontario, Canada

^f^ Department of Medicine (Geriatrics), School of Medicine, University College Cork, Cork, Ireland

^g^ Department of Geriatric Medicine, Cork University Hospital, Wilton, Cork, Ireland.

^h^ Department of Clinical and Molecular Sciences, Università Politecnica delle Marche, Ancona, Italy

^i^ Geriatria, Accettazione geriatrica e Centro di Ricerca per l’invecchiamento, IRCCS INRCA, Ancona, Italy

^j^ Department of Geriatrics, Orthopedics and Rheumatology, Università Cattolica del Sacro Cuore, Rome, Italy

^k^ Center of Aging, Fondazione Policlinico Universitario Gemelli IRCCS, Rome, Italy

^l^ Department of Internal Medicine and Paediatrics, Ghent University, Ghent, Belgium

^m^ Pharmaceutical Care Research Group, School of Pharmacy, University College Cork, Cork, Ireland

^n^ Leslie Dan Faculty of Pharmacy, University of Toronto, Toronto, Ontario, Canada

^o^ Institute for Better Health, Trillium Health Partners, Toronto, Ontario, Canada

^p^ Women's College Research Institute, Women's College Hospital, Toronto, Ontario, Canada

^q^ Department of Medicine, Division of Geriatrics, Maccabi Healthcare Services, Tel Aviv, Israel

^r^ Hadassah Medical Center and Faculty of Medicine, Hebrew University of Jerusalem, Jerusalem, Israel

^s^ Amsterdam UMC Location University of Amsterdam, Internal Medicine, Section of Geriatric Medicine, Amsterdam, The Netherlands

^t^ Amsterdam Public Health Research Institute, Amsterdam, The Netherlands

^u^ Department of Clinical Pharmacology, Royal North Shore Hospital, Sydney, Australia

^v^ Northern Clinical School, Faculty of Medicine and Health, University of Sydney, Sydney, Australia

^w^ Division of Geriatric Medicine, UMass Chan Medical School, Worcester, Massachusetts, USA

**Corresponding Author**: Paula Rochon, Women’s Age Lab, Women’s College Hospital, RTO/ERO Chair in Geriatric Medicine, University of Toronto, 76 Grenville Street, Toronto, Ontario, Canada M5S 1B2

Email: [paula.rochon@wchospital.ca](mailto:paula.rochon@wchospital.ca)

Twitter: @rochonpaula

ORCID: <https://orcid.org/0000-0002-5973-4151>

**Table of Contents**

eTable 1. Description of Project Conveners and Panelists

eTable 2. Defining a Potentially Inappropriate and Potentially Appropriate Prescribing Cascade

eTable 3. Guidance on Conducting and Reporting Delphi Studies (CREDES) Checklist

# eTables 4a-4j: List of Prescribing Cascades in Questionnaire Round 1 by Physiologic System

# eTable 4a: Cardiovascular System

# eTable 4b: Central Nervous System

# eTable 4c: Coagulation System

# eTable 4d: Endocrine System

# eTable 4e: Gastrointestinal System

# eTable 4f. Miscellaneous System

# eTable 4g. Musculoskeletal System

# eTable 4h. Respiratory System

# eTable 4i. Urogenital System

# eTable 4j. Visual System

# eTable 5: The Summary of Panelists' Responses of all Prescribing Cascades in Questionnaire Round 1 by Physiologic System

# eTable 6: The Summary of Panelists' Responses of all Prescribing Cascades in Questionnaire Round 2 by Physiologic System

# eTable 7: The Summary of Panelists' Responses of all Prescribing Cascades in Discussion Round by Physiologic System

# eAppendix 1: REDCap Platform Description

eAppendix 2: Rationale for further refinements

# eReference

# **eTable 1. Description of Project Conveners and Panelists**

| **Study Conveners** | **Profession** | **Institution** | **Country** | **Sex** | **iKascade team** |
| --- | --- | --- | --- | --- | --- |
| Paula A. Rochon | Geriatrician | Women’s Age Lab, Women’s College Hospital, University of Toronto | Canada | F | Yes |
| Jerry H. Gurwitz | Geriatrician | UMass Chan Medical School, Worcester, Massachusetts | USA | M | Yes |
|  |  |  |  |  |  |
| **Panelists** | **Profession** |  |  |  |  |
| Denis O'Mahony | Geriatrician | Department of Medicine, University College Cork, Cork University Hospital | Ireland | M | Yes |
| Antonio Cherubini | Geriatrician | IRCCS INRCA and Università Politecnica delle Marche, Ancona, Italy | Italy | M | Yes |
| Christina E. Reppas-Rindlisbacher | Geriatrician | University of Toronto | Canada | F | No |
| Donna Zwas | Cardiologist | Hadassah Medical Center Heart Institute, Jerusalem, Israel | Israel | F | Yes |
| Graziano Onder | Geriatrician | Italian National Institute of Health, Rome, Italy | Italy | M | Yes |
| Kieran Dalton | Pharmacist | School of Pharmacy, University College Cork | Ireland | M | No |
| Lisa M. McCarthy | Pharmacist | Trillium Health Partners (TRIU), University of Toronto | Canada | F | Yes |
| Mirko Petrovic | Geriatrician | Ghent University, Belgium | Belgium | M | Yes |
| Nathan M. Stall | Geriatrician | Women’s Age Lab, Women’s College Hospital, Mount Sinai Hospital, University of Toronto | Canada | M | Yes |
| Nathalie van der Velde | Geriatrician | University of Amsterdam; Amsterdam Public Health Research Institute | Netherlands | F | No |
| Sarah N. Hilmer | Geriatrician/ Clinical Pharmacologist | Royal North Shore Hospital; Northern Clinical School, Faculty of Medicine and Health, University of Sydney | Australia | F | No |
| Shelley A. Sternberg | Geriatrician | Maccabi Healthcare Services, Modiin, Israel | Israel | F | Yes |

**Sex - M: Male; F: Female**

# **eTable 2. Defining a Potentially Inappropriate and Potentially Appropriate Prescribing Cascade**

| **Example of a Potentially INAPPROPRIATE Prescribing Cascade (PIPC)** |
| --- |
| The example selected to illustrate a PIPC was the calcium channel blocker (CCB) to peripheral edema to diuretic prescribing cascade^1^. This prescribing cascade occurs when an individual is started on a calcium-channel blocker (Drug A), subsequently develops a drug-induced adverse event of peripheral edema that is typically misinterpreted as a new medical condition resulting in a diuretic (Drug B) being initiated to treat this adverse event^2,3^. This prescribing cascade is potentially inappropriate because the peripheral edema is an adverse event associated with the CCB (Drug A) and not a new medical condition. CCB-related edema stems from fluid redistribution, not fluid overload; therefore, treating euvolemic individuals with a diuretic may increase their risk of additional harm. Additionally, alternative therapies to CCBs are available. By recognizing that the adverse drug event of peripheral edema is likely related to Drug A, the need for the diuretic therapy can be re-evaluated and, in some cases, discontinued (with the dose of the calcium channel blocker modified or an alternative therapy chosen).  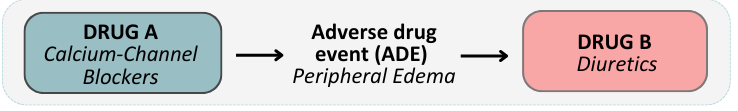 |
| **Example of a Potentially APPROPRIATE Prescribing Cascade** |
| The example selected to illustrate a potentially appropriate prescribing cascade was the opioid to constipation to laxative therapy sequence. This potentially appropriate prescribing cascade occurs when an individual is prescribed an opioid (Drug A) for an appropriate indication, and subsequently develops constipation (a well-known adverse event associated with opioid therapy), and a laxative is prescribed (Drug B) in accordance with clinical guidelines^4^. By starting the laxative (Drug B), the adverse event arising from the opioid (Drug A) specifically, constipation is appropriately managed.  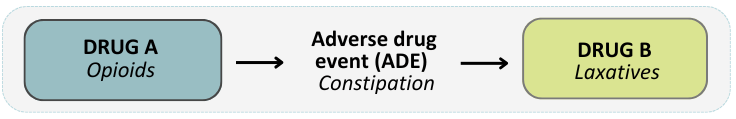 |

**eTable 3. Guidance on Conducting and Reporting Delphi Studies (CREDES) Checklist**

| **Guidance on Conducting and Reporting Delphi Studies (CREDES) Checklist** |  |
| --- | --- |
| **Rationale for Delphi Technique** |  |
| ***Justification***. The choice of the Delphi technique as a method of systematically  collating expert consultation and building consensus needs to be well justified. |  |
| **Planning and Design** |  |
| ***Planning and process***. The Delphi technique is a flexible method and can be adjusted to the respective research aims and purposes. Any modifications should be justified by a rationale and be applied systematically and rigorously |  |
| ***Definition of consensus***. Unless not reasonable due to the explorative nature of the study, an a priori criterion for consensus should be defined. This includes a clear and transparent guide for action on (a) h*ow to proceed with certain items or topics in the next survey round,* (b) the required threshold to terminate the Delphi process and (c) procedures to be followed when consensus is (not) reached after one or more iterations |  |
| **Study Conduct** |  |
| ***Informational input***. All material provided to the expert panel at the outset of the project and throughout the Delphi process should be carefully reviewed and piloted in advance in order to examine the effect on experts’ judgements and to prevent bias |  |
| ***Prevention of bias***. Researchers need to take measures to avoid directly or indirectly influencing the experts’ judgements. If one or more members of the research team have a conflict of interest, entrusting an independent researcher with the main coordination of the Delphi study is advisable |  |
| ***Interpretation and processing of results****.* Consensus does not necessarily imply the ‘correct’ answer or judgement; (non)consensus and stable disagreement provide informative insights and highlight differences in perspectives concerning the topic in question |  |
| ***External validation****.* It is recommended to have the final draft of the resulting guidance reviewed and approved by an external board or authority before publication and dissemination |  |
| **Reporting** |  |
| ***Purpose and rationale****.* The purpose of the study should be clearly defined and demonstrate the appropriateness of the use of the Delphi technique as a method to achieve the research aim. A rationale for the choice of the Delphi technique as the most suitable method needs to be provided | Page 6 |
| ***Expert panel****.* Criteria for the selection of experts and transparent information on recruitment of the expert panel | Page 9 |
| ***Description of the methods****.* The methods employed need to be comprehensible; this includes information on preparatory steps, piloting of material and survey instruments, design of the survey instrument(s), the number and design of survey.  Rounds (anticipated 2), *methods of data analysis, processing and synthesis of experts’ responses* inform the subsequent survey round and methodological decisions taken by the research team throughout the process | Page6-11 |
| ***Procedure****.* Flow chart to illustrate the stages of the Delphi process, including a  preparatory phase, the actual ‘Delphi rounds’, interim steps of data processing and analysis, and concluding steps | Page 21 |
| ***Definition and attainment of consensus****.* It needs to be comprehensible to the reader how consensus was achieved throughout the process, including strategies to deal with non-consensus | Page 10-11 |
| ***Results****.* Reporting of results for each round separately is highly advisable in order to make the evolving of consensus over the rounds transparent. This includes figures showing the average group response, changes between rounds, as well as any modifications of the survey instrument such as deletion, addition or modification of survey items based on previous rounds | Page 11-12,21 |
| ***Discussion of limitations****.* Reporting should include a critical reflection of potential limitations and their impact of the resulting guidance | Page 13 |
| ***Adequacy of conclusions****.* The conclusions should adequately reflect the outcomes of the Delphi study with a view to the scope and applicability of the resulting practice guidance | Page 13 |
| Publication and dissemination. The resulting guidance should be clearly identifiable from the publication, including recommendations for transfer into practice and implementation. A dissemination plan should include endorsement of the guidance by professional associations and health care authorities to facilitate implementation | Page 13 |

# **eTables 4a-4j: List of Prescribing Cascades in Questionnaire Round 1 by Physiologic System**

# eTable 4a: Cardiovascular System

| **Drug A: Initial Drug Therapy** | **Adverse Drug Event** | **Drug B: New Drug Therapy** | **Source Type and References** |
| --- | --- | --- | --- |
| Angiotensin converting enzyme inhibitor | Cough | Cough remedy | PSSA^5^  PSSA^6^  PSSA^7^ |
|  | Urinary Tract Infections | Antibiotic | PSSA^8^  PSSA^9^ |
| Antihypertensive | Orthostatic hypotension/dizziness | Antiemetic | Clinical Review^10^ |
| Beta blocker (particularly lipophilic e.g., propranolol) | Depression | Antidepressant | PSSA^11^  Retrospective Cohort Study^12^  Retrospective Cohort Study^13^ |
|  | Erectile dysfunction | Phosphodiesterase-5 inhibitors, alprostadil | PSSA^14^ |
| Calcium channel blocker | Peripheral edema | Diuretic | Retrospective Cohort Study^15^  PSSA^16^  PSSA^17^ |
|  | Constipation | Laxative | Retrospective Cohort Study^18^ |
| Diuretic | Gout or Hyperuricemia | Anti-gout agent | Retrospective Cohort Study^19^ |
|  | Hypokalemia | Potassium supplement | PSSA^20^  Clinical Review^21^ |
|  | Urinary incontinence | Overactive bladder medication | PSSA^22^ |
| HMG Co-A reductase inhibitor (statin) | Myalgia/myositis | Pain reliever | PSSA^20^  PSSA^23^  PSSA^24^ |
|  |  | Mineral supplement | Prospective Cohort Study^25^ |
|  |  | Quinine sulfate | PSSA^24^  PSSA^20^ |
|  | Insomnia | Sleep agent | Retrospective Cohort Study^26^ |
|  | Urinary Incontinence | Overactive bladder medication | PSSA^27^  PSSA^9^ |
|  | Depression | Antidepressant | PSSA^9^  PSSA^28^ |
|  | Skin soft tissue infection | Antibiotic | PSSA^29^  PSSA^9^ |
| Amiodarone | Hypothyroidism | Thyroid Hormone | PSSA^30^  PSSA^20^  Retrospective Cohort Study^31^ |
|  |  | Anti-thyroid agent | Case Report^32^ |
| Digoxin | Nausea | Antiemetic | PSSA^33^ |
| Flunarizine | Depression | Antidepressant | PSSA^34^ |
| Midodrine | Hypertension | Antihypertensive | Case Report^35^ |

PSSA: prescription sequence symmetry analysis;

HMG-CoA reductase inhibitor: hydroxymethylglutaryl-coenzyme A reductase inhibitor

# eTable 4b: Central Nervous System

| **Drug A: Initial Drug Therapy** | **Adverse Drug Event** | **Drug B: New Drug Therapy** | **Source Type and References** |
| --- | --- | --- | --- |
| Anticonvulsant | Rash | Topical corticosteroid | PSSA^36^ |
|  | Nausea | Antiemetic | PSSA^33^  PSSA^36^ |
|  | Hypothyroidism | Thyroid Hormone | Case-control Study^37^  PSSA^38^ |
| Antiepileptic drug | Hypothyroidism | Levothyroxine | PSSA^38^ |
| Gabapentinoid | Peripheral edema | Diuretic | Retrospective Cohort Study^39^  Clinical Review^21^ |
| Antipsychotic | Extrapyramidal symptoms | Beta-blocker | Clinical Review^40^ |
|  |  | Antiparkinsonian agent | PSSA^41^  Case-control Study^42^  Retrospective Cohort Study^43^ |
|  |  | Anti-tremor antimuscarinic | PSSA^44^  Cross-sectional Study^45^ |
|  | Akathisia or tardive movements | Sedative | Clinical Review^46^ |
|  | Arrhythmia | Antiarrhythmic | PSSA^44^ |
|  | Hyperglycemia | Antihyperglycemic | PSSA^47^  PSSA^48^ |
|  | Dyslipidemia | HMG Co-A reductase inhibitor (statin) | PSSA^49^ |
|  | Osteoporosis | Bisphosphonate | PSSA^50^ |
| Benzodiazepine | Cognitive impairment | Cholinesterase Inhibitors or Memantine | PSSA^51^  PSSA^52^ |
| Dopaminergic Antiparkinsonian agent | Psychotic symptoms, hallucinations | Antipsychotic | Cross-sectional Study^53^  Clinical Review^54^ |
| Cholinesterase Inhibitor (and memantine) | Urinary incontinence | Overactive bladder medication | Retrospective Cohort Study^55^  Retrospective Cohort Study^56^ |
|  | Insomnia | Sleep agent | PSSA^57^ |
|  | Gastrointestinal upset | Antiemetic | PSSA^57^ |
|  |  | Bismuth subsalicylate | Case Report^58^ |
|  | Diarrhea | Anti-diarrheal | PSSA^57^ |
|  | Gastritis/gastric ulcer/GI bleed | Gastroprotective agent | Retrospective Cohort Study^59^ |
|  | Rhinorrhea | Antihistamine | Retrospective Cohort Study^60^  Case Report^61^ |
|  | Chronic airways disorders | Antibacterial and corticosteroid | PSSA^62^ |
| SSRI/SNRI | Urinary Incontinence | Overactive bladder medication | Retrospective Cohort Study^63^ |
|  | Restless leg syndrome | Dopamine agonist | PSSA^64^ |
| Tricyclic Antidepressant | Cognitive Impairment | Cholinesterase Inhibitors or Memantine | Clinical Review^21^ |
|  | Constipation | Laxative | Clinical Review^21^ |
|  | Urinary Incontinence | Overactive bladder medication | Clinical Review^65^ |
| Anticholinergic agent | Gastritis/gastric ulcer/GI bleed | Gastroprotective agent | Retrospective Cohort Study^66^ |
| Antiparkinson drug | Depression | Antidepressant | Retrospective Cohort Study^67^ |
| Lithium | Extrapyramidal symptoms | Antiparkinsonian agent | Retrospective Cohort Study^68^  Retrospective Cohort Study^67^ |
|  |  | Beta-blocker | Case Report^69^ |
|  | Hypothyroidism | Thyroxine | PSSA^20^  Case-control Study^37^ |
| Varenicline | Depression | Antidepressant | Retrospective Cohort Study^70^  PSSA^71^ |
| Venlafaxine | Hypertension (dose-related) | Antihypertensive | Case Report^72^  Meta-analysis Study^73^ |
|  | Tremor | Benzodiazepine | Clinical Review^21^ |

PSSA: prescription sequence symmetry analysis

HMG-CoA reductase inhibitor: hydroxymethylglutaryl-coenzyme A reductase inhibitor

GI: gastrointestinal

SSRI/SNRI: selective serotonin reuptake inhibitor /serotonin and norepinephrine reuptake inhibitor

# eTable 4c: Coagulation System

| **Drug A: Initial Drug Therapy** | **Adverse Drug Event** | **Drug B: New Drug Therapy** | **Source Type and References** |
| --- | --- | --- | --- |
| Anticoagulant | Gastritis/gastric ulcer/GI bleed | Gastroprotective agent | PSSA^74^ |
|  | Osteoporosis | Bisphosphonate | PSSA^75^ |
| Antiplatelet | Gastritis/gastric ulcer/GI bleed | Gastroprotective agent | PSSA^76^ |
| Direct oral anticoagulant | Depression | Antidepressant | PSSA^74^ |
| Acetylsalicylic acid | Gastritis/gastric ulcer/GI bleed | Gastroprotective agent | PSSA^77^ |
| Dabigatran | Gastritis/gastric ulcer/GI bleed | Gastroprotective agent | PSSA^74^ |

PSSA: prescription sequence symmetry analysis

GI: gastrointestinal

# eTable 4d: Endocrine System

| **Drug A: Initial Drug Therapy** | **Adverse Drug Event** | **Drug B: New Drug Therapy** | **Source Type and References** |
| --- | --- | --- | --- |
| Dipeptidyl Peptidase 4 (DPP-4) Inhibitor | Joint pain | Non-steroidal anti-inflammatory drug | Retrospective Cohort Study^78^ |
| Sodium-glucose cotransporter-2 (SGLT-2) inhibitor | Urinary tract infections | Antibiotic | Retrospective Cohort Study^79^  Retrospective Cohort Study^80^ |
|  | Mycotic genital infections | Antifungal | Retrospective Cohort Study^80^  PSSA^81^ |
|  | Gout | Anti-gout agent | Retrospective Cohort Study^82^ |
| Metformin | Diarrhea | Antidiarrheal agent | Case Report^83^ |
|  | Vitamin B12 deficiency | Vitamin B12 supplement | Cross-sectional Study^84^ |
| Pioglitazone or Rosiglitazone | Oedema | Furosemide | PSSA^85^ |
| Rosiglitazone | Heart failure | Furosemide | Case Report^86^ |

PSSA: prescription sequence symmetry analysis

# eTable 4e: Gastrointestinal System

| **Drug A: Initial Drug Therapy** | **Adverse Drug Event** | **Drug B: New Drug Therapy** | **Source Type and References** |
| --- | --- | --- | --- |
| Proton pump inhibitor | Clostridium difficile infection | Antibiotic | PSSA^87^ |
|  | Osteoporosis, fractures | Vitamin supplement | Retrospective Cohort Study^66^ |
|  | Vitamin or Mineral Deficiency | Vitamin or Mineral Supplement | Clinical Review^21^  Case Report^88^ |
|  | Dementia | Anit-dementia medication | PSSA^89^ |
|  | COPD | Muscarinic antagonist or beta-2 agonist for treatment COPD | PSSA^90^ |
| Anticholinergic antiemetic | Urinary retention | Alpha 1 receptor blocker | Clinical Review^91^ |
| Antidopaminergic antiemetic | Extrapyramidal symptoms | Antiparkinsonian agent | Case-control Study^92^ |
| Laxative | Diarrhea | Antidiarrheal agent | Clinical Review^21^ |
| Ranitidine | Heart failure | Furosemide | PSSA^93^  PSSA^94^ |

PSSA: prescription sequence symmetry analysis

COPD: chronic obstructive pulmonary disease

# eTable 4f. Miscellaneous System

| **Drug A: Initial Drug Therapy** | **Adverse Drug Event** | **Drug B: New Drug Therapy** | **Source Type and References** |
| --- | --- | --- | --- |
| Antibiotic | Clostridium difficile infection | Vancomycin, metronidazole, fidamoxicine | Clinical Review^10^ |
|  | Nausea | Antiemetic | PSSA^33^ |
| Corticosteroid | Insomnia | Sleep agent | Focused Review^95^ |
|  | Psychosis | Antipsychotic | Cases Review^96^ |
|  | Hypertension | Antihypertensive | Retrospective Cohort Study^97^ |
| Carbapenem | Seizures | Anticonvulsant | Case Report^32^ |
| Ciprofloxacin | Delirium | Antipsychotic | Clinical Review^21^ |
| Erythromycin | Arrhythmia | Antiarrhythmic | PSSA^98^ |
| Fludrocortisone | Hypertension | Antihypertensive | Review^99^ |
| Isotretinoin | Depression | Antidepressant | Case-crossover Study^100^ |
| Acitretin | Vulvo-vaginal candidiasis | Antifungal | PSSA^101^ |
| Iron Supplement | Constipation | Laxative | Cross-sectional Study^102^ |

PSSA: prescription sequence symmetry analysis

# eTable 4g. Musculoskeletal System

| **Drug A: Initial Drug Therapy** | **Adverse Drug Event** | **Drug B: New Drug Therapy** | **Source Type and References** |
| --- | --- | --- | --- |
| Bisphosphonate | Gastritis | Gastroprotective agent | Case-control Study^103^ |
| NSAID | Gastritis/gastric ulcer/ gastrointestinal bleed | Gastroprotective agent | Case-control Study^104^ |
|  | Nausea | Antiemetic | PSSA^105^  PSSA^33^ |
|  | Hypertension | Antihypertensive | Case-control Study^106^ |
|  | Worsening of heart failure | Digoxin | Case-control Study^107^ |
| Opioid | Nausea/dizziness | Antiemetic | PSSA^33^  PSSA^105^ |
|  | Depression | Antidepressant | Clinical Review^108^ |

PSSA: prescription sequence symmetry analysis

NSAID: Non-steroidal anti-inflammatory drug

# eTable 4h. Respiratory System

| **Drug A: Initial Drug Therapy** | **Adverse Drug Event** | **Drug B: New Drug Therapy** | **Source Type and References** |
| --- | --- | --- | --- |
| Inhaled Corticosteroid | Oral candidiasis | Antifungal | PSSA^109^  PSSA^110^  PSSA^111^ |
| Montelukast | Depression | Antidepressant | PSSA^112^  PSSA^113^ |

PSSA: prescription sequence symmetry analysis

# eTable 4i. Urogenital System

| **Drug A: Initial Drug Therapy** | **Adverse Drug Event** | **Drug B: New Drug Therapy** | **Source Type and References** |
| --- | --- | --- | --- |
| Alpha-1 receptor blocker | Orthostatic hypotension, dizziness | Vestibular suppressant | Clinical Review^114^ |
| Urinary anticholinergic | Dry mouth | Saliva substitute | Prospective Cohort Study^115^ |
| 5-alpha-reductase inhibitor | Depression | Antidepressant | PSSA^116^ |

PSSA: prescription sequence symmetry analysis

# eTable 4j. Visual System

| **Drug A: Initial Drug Therapy** | **Adverse Drug Event** | **Drug B: New Drug Therapy** | **Source Type and References** |
| --- | --- | --- | --- |
| Brinzolamide | Heart failure | Furosemide | PSSA^93^  PSSA^94^  Case-control Study^117^ |
| Latanoprost | Heart failure | Furosemide | PSSA^93^  PSSA^94^  Case-control Study^117^ |

PSSA: prescription sequence symmetry analysis

# **eTable 5: Summary of Panelists' Responses to all Prescribing Cascades in Questionnaire Round 1 by Physiologic System**

| **Prescribing Cascades in Round 1 Delphi Survey (n=107)** | **The following sequences represent a potentially inappropriate prescribing cascade. Choose your level of agreement (Round 1)** | | |
| --- | --- | --- | --- |
|  | **Reponses from 12 panel members** | **Percentage of Agree/Strongly agree** | **Decision on the prescribing cascade** |
| **1. Cardiovascular System** | | | |
| 1.1 Angiotensin converting enzyme inhibitor ⇒ Cough ⇒ Cough remedy |  | 92% | Included |
| 1.2 Angiotensin converting enzyme inhibitor ⇒ Urinary Tract Infections ⇒ Antibiotic |  | 33% | Excluded |
| 1.3 Antihypertensive ⇒ Orthostatic hypotension/dizziness ⇒ Antiemetic |  | 83% | Included |
| 1.4 Beta blocker (particularly lipophilic e.g., propranolol) ⇒ Depression ⇒ Antidepressant |  | 92% | Included |
| 1.5 Beta blocker⇒ Erectile dysfunction ⇒ Phosphodiesterase-5 inhibitor, alprostadil |  | 75% | Included |
| 1.6 Calcium channel blocker ⇒ Peripheral edema ⇒ Diuretic |  | 100% | Included |
| 1.7 Calcium Channel Blocker ⇒ Constipation ⇒ Laxative |  | 83% | Included |
| 1.8 Diuretic ⇒ Gout or Hyperuricemia ⇒ Anti-gout agent (e.g., allopurinol) |  | 83% | Included |
| 1.9 Diuretic ⇒ Hypokalemia ⇒ Potassium supplement |  | 33% | Excluded |
| 1.10 Diuretic ⇒ Urinary incontinence ⇒ Overactive bladder medication |  | 100% | Included |
| 1.11 Hydroxymethylglutaryl-coenzyme A (HMG Co-A) reductase inhibitor (statin) ⇒ Myalgia/myositis ⇒ Pain reliever |  | 100% | Included |
| 1.12 Hydroxymethylglutaryl-coenzyme A (HMG Co-A) reductase inhibitor (statin) ⇒ Myalgia/myositis ⇒ Mineral supplement |  | 67% | Round 2 |
| 1.13 Hydroxymethylglutaryl-coenzyme A (HMG Co-A) reductase inhibitor (statin) ⇒ Insomnia ⇒ Sleep agent |  | 50% | Round 2 |
| 1.14 Hydroxymethylglutaryl-coenzyme A (HMG Co-A) reductase inhibitor (statin) ⇒ Urinary Incontinence ⇒ Overactive bladder medication |  | 33% | Excluded |
| 1.15 Hydroxymethylglutaryl-coenzyme A (HMG Co-A) reductase inhibitor (statin) ⇒ Myalgia/myositis ⇒ Quinine sulfate |  | 83% | Included |
| 1.16 Hydroxymethylglutaryl-coenzyme A (HMG Co-A) reductase inhibitor (statin) ⇒ Depression ⇒ Antidepressant |  | 50% | Round 2 |
| 1.17 Hydroxymethylglutaryl-coenzyme A (HMG Co-A) reductase inhibitor (statin) ⇒ Skin soft tissue infection ⇒ Antibiotic |  | 33% | Excluded |
| 1.18 Amiodarone ⇒ Hypothyroidism ⇒ Thyroid Hormone |  | 58% | Round 2 |
| 1.19 Amiodarone ⇒ Hyperthyroidism ⇒ Anti-thyroid agent |  | 50% | Round 2 |
| 1.20 Digoxin ⇒ Nausea ⇒ Antiemetic |  | 83% | Included |
| 1.21 Flunarizine ⇒ Depression ⇒ Antidepressant |  | 67% | Round 2 |
| 1.22 Midodrine ⇒ Hypertension ⇒ Antihypertensive |  | 100% | Included |
| **2. Central Nervous System** | | | |
| 2.1 Anticonvulsant ⇒ Rash ⇒ Topical corticosteroid |  | 75% | Included |
| 2.2 Anticonvulsant ⇒ Nausea ⇒ Antiemetic |  | 83% | Included |
| 2.3 Anticonvulsant ⇒ Hypothyroidism ⇒ Thyroid Hormone |  | 58% | Round 2 |
| 2.4 Antiepileptic drug ⇒ Hypothyroidism ⇒ Levothyroxine |  | 67% | Round 2 |
| 2.5 Gabapentinoid ⇒ Peripheral edema ⇒ Diuretic |  | 100% | Included |
| 2.6 Antipsychotic ⇒ Extrapyramidal symptoms ⇒ Beta-blocker |  | 67% | Round 2 |
| 2.7 Antipsychotic ⇒ Extrapyramidal symptoms ⇒ Antiparkinsonian agent |  | 92% | Included |
| 2.8 Antipsychotic ⇒ Extrapyramidal symptoms ⇒ Anti-tremor antimuscarinic |  | 83% | Included |
| 2.9 Antipsychotic ⇒ Akathisia or tardive movements ⇒ Sedative |  | 92% | Included |
| 2.10 Antipsychotic ⇒ Arrhythmia ⇒ Antiarrhythmic |  | 83% | Included |
| 2.11 Antipsychotic ⇒ Hyperglycemia ⇒ Antihyperglycemic |  | 75% | Included |
| 2.12 Antipsychotic ⇒ Dyslipidemia ⇒ Hydroxymethylglutaryl-coenzyme A (HMG Co-A) reductase inhibitor (statin) |  | 67% | Round 2 |
| 2.13 Antipsychotic ⇒ Osteoporosis ⇒ Bisphosphonate |  | 58% | Round 2 |
| 2.14 Benzodiazepine ⇒ Cognitive impairment ⇒ Cholinesterase Inhibitor or Memantine |  | 100% | Included |
| 2.15 Dopaminergic Antiparkinsonian agent ⇒ Psychotic symptoms, hallucinations ⇒ Antipsychotic |  | 100% | Included |
| 2.16 Cholinesterase Inhibitor (and memantine) ⇒ Urinary incontinence ⇒ Overactive bladder medication |  | 100% | Included |
| 2.17 Cholinesterase Inhibitor (and memantine) ⇒ Insomnia ⇒ Sleep agent |  | 83% | Included |
| 2.18 Cholinesterase Inhibitor (and memantine) ⇒ Gastrointestinal upset ⇒ Anti-emetic |  | 92% | Included |
| 2.19 Cholinesterase Inhibitor (and memantine) ⇒ Gastrointestinal upset ⇒ Bismuth subsalicylate |  | 92% | Included |
| 2.20 Cholinesterase Inhibitor (and memantine) ⇒ Diarrhea ⇒ Anti-diarrheal |  | 100% | Included |
| 2.21 Cholinesterase Inhibitor (and memantine) ⇒ Gastritis/gastric ulcer/ gastrointestinal bleed ⇒ Gastroprotective agent |  | 58% | Round 2 |
| 2.22 Cholinesterase Inhibitor (and memantine) ⇒ Rhinorrhea ⇒ Antihistamine |  | 83% | Included |
| 2.23 Cholinesterase Inhibitor⇒ Chronic airways disorders ⇒ Antibacterial and corticosteroid |  | 58% | Round 2 |
| 2.24 Selective serotonin reuptake inhibitor / Serotonin and norepinephrine reuptake inhibitor (SSRI/ SNRI) ⇒ Urinary Incontinence ⇒ Overactive bladder medication |  | 83% | Included |
| 2.25 Selective serotonin reuptake inhibitor / Serotonin and norepinephrine reuptake inhibitor (SSRI/SNRI) ⇒ Restless leg syndrome ⇒ Dopamine agonist |  | 67% | Round 2 |
| 2.26 Tricyclic Antidepressant ⇒ Cognitive Impairment ⇒ Cholinesterase Inhibitor or Memantine |  | 100% | Included |
| 2.27 Tricyclic Antidepressant ⇒ Constipation ⇒ Laxative |  | 83% | Included |
| 2.28 Tricyclic Antidepressant ⇒ Urinary Incontinence ⇒ Overactive bladder medication |  | 75% | Included |
| 2.29 Anticholinergic agent ⇒ Gastritis/gastric ulcer/ gastrointestinal bleed ⇒ Gastroprotective agent |  | 42% | Excluded |
| 2.30 Antiparkinson drug⇒ Depression ⇒ Antidepressant |  | 42% | Excluded |
| 2.31 Lithium ⇒ Extrapyramidal symptoms ⇒ Antiparkinsonian agent |  | 92% | Included |
| 2.32 Lithium ⇒ Extrapyramidal symptoms ⇒ Beta-blocker |  | 67% | Round 2 |
| 2.33 Lithium ⇒ Hypothyroidism ⇒ Thyroxine |  | 58% | Round 2 |
| 2.34 Varenicline ⇒ Depression ⇒ Antidepressant |  | 67% | Round 2 |
| 2.35 Venlafaxine ⇒ Hypertension (dose-related) ⇒ Antihypertensive |  | 92% | Included |
| 2.36 Venlafaxine ⇒ Tremor ⇒ Benzodiazepine |  | 83% | Included |
| **3. Coagulation System** |  |  |  |
| 3.1 Anticoagulant ⇒ Gastritis/gastric ulcer/ gastrointestinal bleed ⇒ Gastroprotective agent |  | 17% | Excluded |
| 3.2 Anticoagulant ⇒ Osteoporosis ⇒ Bisphosphonate |  | 50% | Round 2 |
| 3.3 Antiplatelet ⇒ Gastritis/gastric ulcer/ gastrointestinal bleed ⇒ Gastroprotective agent |  | 17% | Excluded |
| 3.4 Direct oral anticoagulant (DOAC) ⇒ Depression ⇒ Antidepressant |  | 42% | Excluded |
| 3.5 Acetylsalicylic acid (ASA) ⇒ Gastritis/gastric ulcer/ gastrointestinal bleed ⇒ Gastroprotective agent |  | 17% | Excluded |
| 3.6 Dabigatran ⇒ Gastritis/gastric ulcer/ gastrointestinal bleed ⇒ Gastroprotective agent |  | 17% | Excluded |
| **4. Endocrine System** |  |  |  |
| 4.1 Dipeptidyl Peptidase 4 (DPP-4) Inhibitor (e.g., sitagliptin, saxagliptin) ⇒ Joint pain ⇒ Nonsteroidal anti-inflammatory drug (NSAID) |  | 75% | Included |
| 4.2 Sodium-glucose cotransporter-2 (SGLT-2) inhibitor ⇒ Urinary tract infections ⇒ Antibiotic |  | 67% | Round 2 |
| 4.3 Sodium-glucose cotransporter-2 (SGLT-2) inhibitor ⇒ Mycotic genital infections ⇒ Antifungal |  | 75% | Included |
| 4.4 Sodium-glucose cotransporter-2 (SGLT-2) inhibitor ⇒ Gout ⇒ Anti-gout agent (e.g., allopurinol) |  | 50% | Round 2 |
| 4.5 Metformin ⇒ Diarrhea ⇒ Antidiarrheal agent (e.g. loperamide, diphenoxylate) |  | 92% | Included |
| 4.6 Metformin ⇒ Vitamin B12 deficiency ⇒ Vitamin B12 supplement |  | 50% | Round 2 |
| 4.7 Pioglitazone or Rosiglitazone ⇒ Oedema ⇒ Furosemide |  | 92% | Included |
| 4.8 Rosiglitazone ⇒ Heart failure ⇒ Furosemide |  | 83% | Included |
| **5. Gastrointestinal System** |  |  |  |
| 5.1 Proton pump inhibitor ⇒ Clostridium difficile infection ⇒ Antibiotic |  | 58% | Round 2 |
| 5.2 Proton pump inhibitor ⇒ Osteoporosis, fractures ⇒ Vitamin supplement |  | 58% | Round 2 |
| 5.3 Proton pump inhibitor ⇒ Vitamin or Mineral Deficiency ⇒ Vitamin or Mineral Supplement |  | 67% | Round 2 |
| 5.4 Proton pump inhibitor ⇒ Dementia ⇒ anit-dementia medication |  | 33% | Excluded |
| 5.5 Proton pump inhibitor ⇒ COPD ⇒ muscarinic antagonist or beta-2 agonist for treatment COPD |  | 50% | Round 2 |
| 5.6 Anticholinergic antiemetic ⇒ Urinary retention ⇒ Alpha 1 receptor blocker |  | 100% | Included |
| 5.7 Antidopaminergic antiemetic ⇒ Extrapyramidal symptoms ⇒ Antiparkinsonian agent |  | 100% | Included |
| 5.8 Laxative ⇒ Diarrhea ⇒ Antidiarrheal agent |  | 100% | Included |
| 5.9 Ranitidine ⇒ Heart failure ⇒ Furosemide |  | 33% | Excluded |
| **6. Miscellaneous System** |  |  |  |
| 6.1 Antibiotic ⇒ Clostridium difficile infection ⇒ Vancomycin, metronidazole, fidamoxicine |  | 17% | Excluded |
| 6.2 Antibiotic ⇒ Nausea ⇒ Antiemetic |  | 25% | Excluded |
| 6.3 Corticosteroid ⇒ Insomnia ⇒ Sleep agent |  | 83% | Included |
| 6.4 Corticosteroid ⇒ Psychosis ⇒ Antipsychotic |  | 92% | Included |
| 6.5 Corticosteroid ⇒ Hypertension ⇒ Antihypertensive |  | 75% | Included |
| 6.6 Carbapenem (e.g., imipenem, meropenem, etrapenem) ⇒ Seizures ⇒ Anticonvulsant |  | 67% | Round 2 |
| 6.7 Ciprofloxacin ⇒ Delirium ⇒ Antipsychotic |  | 67% | Round 2 |
| 6.8 Erythromycin ⇒ Arrhythmia ⇒ Antiarrhythmic |  | 67% | Round 2 |
| 6.9 Fludrocortisone ⇒ Hypertension ⇒ Antihypertensive |  | 83% | Included |
| 6.10 Isotretinoin ⇒ Depression ⇒ Antidepressant |  | 58% | Round 2 |
| 6.11 Acitretin ⇒ Vulvo-vaginal candidiasis ⇒ Antifungal |  | 75% | Included |
| 6.12 Iron Supplement ⇒ Constipation ⇒ Laxative |  | 75% | Included |
| **7. Musculoskeletal System** |  |  |  |
| 7.1 Bisphosphonate ⇒ Gastritis ⇒ Gastroprotective agent |  | 92% | Included |
| 7.2 NSAID ⇒ Gastritis/gastric ulcer/ gastrointestinal bleed ⇒ Gastroprotective agent |  | 100% | Included |
| 7.3 Nonsteroidal anti-inflammatory drug (NSAID) ⇒ Nausea ⇒ Antiemetic |  | 83% | Included |
| 7.4 Nonsteroidal anti-inflammatory drug (NSAID) ⇒ Hypertension ⇒ Antihypertensive |  | 100% | Included |
| 7.5 Nonsteroidal anti-inflammatory drug (NSAID) ⇒ Worsening of heart failure ⇒ Digoxin |  | 92% | Included |
| 7.6 Opioid ⇒ Nausea/dizziness ⇒ Antiemetic |  | 58% | Round 2 |
| 7.7 Opioid ⇒ Depression ⇒ Antidepressant |  | 75% | Included |
| **8. Respiratory System** |  |  |  |
| 8.1 Inhaled Corticosteroid ⇒ Oral candidiasis ⇒ Antifungal e.g., nystatin |  | 42% | Excluded |
| 8.2 Montelukast ⇒ Depression ⇒ Antidepressant |  | 50% | Round 2 |
| **9. Urogenital System** |  |  |  |
| 9.1 Alpha 1 receptor blocker ⇒ Orthostatic hypotension, dizziness ⇒ Vestibular suppressant |  | 92% | Included |
| 9.2 Urinary anticholinergic ⇒ Dry mouth ⇒ Saliva substitute |  | 67% | Round 2 |
| 9.3 5-alpha-reductase inhibitor ⇒ Depression ⇒ Antidepressant |  | 42% | Excluded |
| **10. Visual System** |  |  |  |
| 10.1 Brinzolamide ⇒ Heart failure ⇒ Furosemide |  | 33% | Excluded |
| 10.2 Latanoprost ⇒ Heart failure ⇒ Furosemide |  | 42% | Excluded |

Note for the criteria for inclusion: If ≥75% panelists agree or strongly agree, the prescribing cascade was included in the PIPC list. If <50% panelists agree or strongly agree, the prescribing cascades are excluded. If 50%-74% panelists agree or strongly agree, the prescribing cascades go to the questionnaire round 2.

# **eTable 6: The Summary of Panelists' Responses of all Prescribing Cascades in Questionnaire Round 2 by Physiologic System**

| **Delphi Survey Round 2 Prescribing Cascades (n=32)** | **The following sequences represent a potentially inappropriate prescribing cascade. Choose your level of agreement (Round 2)** | | |
| --- | --- | --- | --- |
|  | **Reponses from 12 panel members** | **Percentage of Agree/Strongly agree** | **Decision on the prescribing cascade** |
| **1. Cardiovascular System** | | | |
| 1.12 Hydroxymethylglutaryl-coenzyme A (HMG Co-A) reductase inhibitor (statin) ⇒ Myalgia/myositis ⇒ Mineral supplement |  | 75% | Included |
| 1.13 Hydroxymethylglutaryl-coenzyme A (HMG Co-A) reductase inhibitor (statin) ⇒ Insomnia ⇒ Sleep agent |  | 75% | Included |
| 1.16 Hydroxymethylglutaryl-coenzyme A (HMG Co-A) reductase inhibitor (statin) ⇒ Depression ⇒ Antidepressant |  | 42% | Excluded |
| 1.18 Amiodarone ⇒ Hypothyroidism ⇒ Thyroid Hormone |  | 42% | Excluded |
| 1.19 Amiodarone ⇒ Hyperthyroidism ⇒ Anti-thyroid agent |  | 42% | Excluded |
| 1.21 Flunarizine ⇒ Depression ⇒ Antidepressant |  | 75% | Included |
| **2. Central Nervous System** |  |  |  |
| 2.3 Anticonvulsant ⇒ Hypothyroidism ⇒ Thyroid Hormones |  | 67% | Discussion Round |
| 2.4 Antiepileptic drug ⇒ Hypothyroidism ⇒ Levothyroxine |  | 67% | Discussion Round |
| 2.6 Antipsychotic ⇒ Extrapyramidal symptoms ⇒ Beta-blocker |  | 75% | Included |
| 2.12 Antipsychotic ⇒ Dyslipidemia ⇒ Hydroxymethylglutaryl-coenzyme A (HMG Co-A) reductase inhibitor (statin) |  | 67% | Discussion Round |
| 2.13 Antipsychotic ⇒ Osteoporosis ⇒ Bisphosphonate |  | 42% | Excluded |
| 2.21 Cholinesterase Inhibitor (and memantine) ⇒ Gastritis/gastric ulcer/ gastrointestinal bleed ⇒ Gastroprotective agent |  | 58% | Discussion Round |
|  |  |  |  |
| 2.23 Cholinesterase Inhibitor⇒ Chronic airways disorders ⇒ Antibacterial and corticosteroid |  | 25% | Excluded |
| 2.25 Selective serotonin reuptake inhibitor / Serotonin and norepinephrine reuptake inhibitor (SSRI/SNRI) ⇒ Restless leg syndrome ⇒ Dopamine agonist |  | 50% | Discussion Round |
| 2.32 Lithium ⇒ Extrapyramidal symptoms ⇒ Beta-blocker |  | 67% | Discussion Round |
| 2.33 Lithium ⇒ Hypothyroidism ⇒ Thyroxine |  | 50% | Discussion Round |
| 2.34 Varenicline ⇒ Depression ⇒ Antidepressant |  | 42% | Excluded |
| **3. Coagulation System** |  |  |  |
| 3.2 Anticoagulant ⇒ Osteoporosis ⇒ Bisphosphonate |  | 33% | Excluded |
| **4. Endocrine System** |  |  |  |
| 4.2 Sodium-glucose cotransporter-2 (SGLT-2) inhibitor ⇒ Urinary tract infections ⇒ Antibiotic |  | 67% | Discussion Round |
| 4.4 Sodium-glucose cotransporter-2 (SGLT-2) inhibitor ⇒ Gout ⇒ Anti-gout agent (e.g., allopurinol) |  | 33% | Excluded |
| 4.6 Metformin ⇒ Vitamin B12 deficiency ⇒ Vitamin B12 supplement |  | 50% | Discussion Round |
| **5. Gastrointestinal System** |  |  |  |
| 5.1 Proton pump inhibitor ⇒ Clostridium difficile infection ⇒ Antibiotic |  | 50% | Discussion Round |
| 5.2 Proton pump inhibitor ⇒ Osteoporosis, fractures ⇒ Vitamin supplement |  | 83% | Included |
| 5.3 Proton pump inhibitor ⇒ Vitamin or Mineral Deficiency ⇒ Vitamin or Mineral Supplement |  | 100% | Included |
| 5.5 Proton pump inhibitor ⇒ Chronic obstructive pulmonary disease (COPD) ⇒ muscarinic antagonist or beta-2 agonist for treatment COPD |  | 25% | Excluded |
| **6. Miscellaneous System** |  |  |  |
| 6.6 Carbapenem (e.g., imipenem, meropenem, etrapenem) ⇒ Seizures ⇒ Anticonvulsant |  | 75% | Included |
| 6.7 Ciprofloxacin ⇒ Delirium ⇒ Antipsychotic |  | 67% | Discussion Round |
|  |  |  |  |
| 6.8 Erythromycin ⇒ Arrhythmia ⇒ Antiarrhythmic |  | 83% | Included |
| 6.10 Isotretinoin ⇒ Depression ⇒ Antidepressant |  | 67% | Discussion Round |
| **7. Musculoskeletal System** |  |  |  |
| 7.6 Opioids ⇒ Nausea/dizziness ⇒ Antiemetic |  | 58% | Discussion Round |
| **8. Respiratory System** |  |  |  |
| 8.2 Montelukast ⇒ Depression ⇒ Antidepressant |  | 50% | Discussion Round |
| **9. Urogenital System** |  |  |  |
| 9.2 Urinary anticholinergic ⇒ Dry mouth ⇒ Saliva substitute |  | 83% | Included |

Note for the criteria for inclusion: If ≥75% panelists agree or strongly agree, the prescribing cascade was included in the PIPC list. If <50% panelists agree or strongly agree, the prescribing cascades are excluded. If 50%-74% panelists agree or strongly agree, the prescribing cascades go to the Discussion Round.

# **eTable 7: The Summary of Panelists' Responses of all Prescribing Cascades in Discussion Round by Physiologic System**

| **Prescribing Cascades Discussed in Discussion Round (n=14)** | **The following sequences represent a potentially inappropriate prescribing cascade. Choose your level of agreement (Consensus Meeting)** | | |
| --- | --- | --- | --- |
|  | **Reponses from 12 panel members** | **Percentage of Agree/Strongly agree** | **Decision on the prescribing cascade** |
| **2. Central Nervous System** | | | |
| 2.3 Anticonvulsant ⇒ Hypothyroidism ⇒ Thyroid Hormone |  | 33% | Excluded |
| 2.4 Antiepileptic drug ⇒ Hypothyroidism ⇒ Levothyroxine |  | 33% | Excluded |
| 2.12 Antipsychotic ⇒ Dyslipidemia ⇒ Hydroxymethylglutaryl-coenzyme A (HMG Co-A) reductase inhibitor (statin) |  | 58% | Excluded |
| 2.21 Cholinesterase Inhibitor (and memantine) ⇒ Gastritis/gastric ulcer/ gastrointestinal bleed ⇒ Gastroprotective agent |  | 33% | Excluded |
| 2.25 Selective serotonin reuptake inhibitor / Serotonin and norepinephrine reuptake inhibitor (SSRI/SNRI) ⇒ Restless leg syndrome ⇒ Dopamine agonist |  | 25% | Excluded |
| 2.32 Lithium ⇒ Extrapyramidal symptoms ⇒ Beta-blocker |  | 50% | Excluded |
| 2.33 Lithium ⇒ Hypothyroidism ⇒ Thyroxine |  | 25% | Excluded |
| **4. Endocrine System** |  |  |  |
| 4.2 Sodium-glucose cotransporter-2 (SGLT-2) inhibitor ⇒ Urinary tract infections ⇒ Antibiotic |  | 25% | Excluded |
| 4.6 Metformin ⇒ Vitamin B12 deficiency ⇒ Vitamin B12 supplement |  | 25% | Excluded |
| **5. Gastrointestinal System** |  |  |  |
| 5.1 Proton pump inhibitor ⇒ Clostridium difficile infection ⇒ Antibiotic |  | 42% | Excluded |
| **6. Miscellaneous System** |  |  |  |
| 6.7 Ciprofloxacin ⇒ Delirium ⇒ Antipsychotic |  | 50% | Excluded |
| 6.10 Isotretinoin ⇒ Depression ⇒ Antidepressant |  | 58% | Excluded |
| **7. Musculoskeletal System** |  |  |  |
| 7.6 Opioid ⇒ Nausea/dizziness ⇒ Antiemetic |  | 50% | Excluded |
| **8. Respiratory System** |  |  |  |
| 8.2 Montelukast ⇒ Depression ⇒ Antidepressant |  | 50% | Excluded |

Note for the criteria for inclusion: If ≥75% panelists agree or strongly agree, the prescribing cascade was included in the PIPC list. If <75% panelists agree or strongly agree, the prescribing cascades are excluded.

# **eAppendix 1: REDCap Platform Description**

REDCap (Research Electronic Data Capture) is a secure, web-based software platform designed to support data capture for research studies, providing 1) an intuitive interface for validated data capture; 2) audit trails for tracking data manipulation and export procedures; 3) automated export procedures for seamless data downloads to common statistical packages; and 4) procedures for data integration and interoperability with external sources.

**eAppendix 2: Rationale for further refinements**

The compehensive list of potentially inappropriate prescribing cascades was reviewed by the study conveners and iKASCADE panel members. During this process, three refinements were made. First, one of the prescribing cascades describing nonsteroidal anti-inflammatory drugs leading to the development of heart failure specified digoxin (Drug B) as a proxy for heart failure treatment. Given that digoxin is no longer a first line heart failure treatment, Drug B was recharacterized from digoxin to the more general term of “heart failure medications”. Second, five prescribing cascades described cholinesterase inhibitors or memantine as Drug A. While both drugs are used in dementia management, the adverse effects of cholinesterase inhibitors and memantine are substantially different and the prescribing cascades were based on adverse effects specific to cholinesterase inhibitors. As such, memantine was removed from being included in Drug A for five of the prescribing cascades. Finally, the pioglitazone or rosiglitazone to edema and furosemide and the rosiglitazone to heart failure and furosemide prescribing cascades were moved from the specific drug B therapy of furosemide to specify the more general drug therapy of diuretics.

# **eReferences – Supplementary Materials**

1. Rochon PA, Austin PC, Normand SL, et al. Association of a calcium channel blocker and diuretic prescribing cascade with adverse events: A population-based cohort study. *Journal of the American Geriatrics Society*. Feb 2024;72(2):467-478. doi:10.1111/jgs.18683

2. Savage RD, Visentin JD, Bronskill SE, et al. Evaluation of a Common Prescribing Cascade of Calcium Channel Blockers and Diuretics in Older Adults With Hypertension. *JAMA internal medicine*. Feb 24 2020;doi:10.1001/jamainternmed.2019.7087

3. Singh S, Cocoros NM, Haynes K, et al. Identifying prescribing cascades in Alzheimer's disease and related dementias: The calcium channel blocker-diuretic prescribing cascade. *Pharmacoepidemiol Drug Saf*. Aug 2021;30(8):1066-1073. doi:10.1002/pds.5230

4. Crockett SD, Greer KB, Heidelbaugh JJ, Falck-Ytter Y, Hanson BJ, Sultan S. American Gastroenterological Association Institute Guideline on the Medical Management of Opioid-Induced Constipation. *Gastroenterology*. Jan 2019;156(1):218-226. doi:10.1053/j.gastro.2018.07.016

5. Vegter S, de Boer P, van Dijk KW, Visser S, de Jong-van den Berg LT. The effects of antitussive treatment of ACE inhibitor-induced cough on therapy compliance: a prescription sequence symmetry analysis. *Drug Saf*. Jun 2013;36(6):435-9. doi:10.1007/s40264-013-0024-z

6. McDonald C. Evaluation of ACE-inhibitor (ACE-I) associated cough using modified prescription sequence analysis (PSA). *Pharmacoepidemiology and Drug Safety*. 1995;4(1):17-22.

7. Vegter S, de Jong-van den Berg LT. Misdiagnosis and mistreatment of a common side-effect--angiotensin-converting enzyme inhibitor-induced cough. *Br J Clin Pharmacol*. Feb 2010;69(2):200-3. doi:10.1111/j.1365-2125.2009.03571.x

8. Pouwels KB, Visser ST, Bos HJ, Hak E. Angiotensin-converting enzyme inhibitor treatment and the development of urinary tract infections: a prescription sequence symmetry analysis. *Drug Saf*. Nov 2013;36(11):1079-86. doi:10.1007/s40264-013-0085-z

9. Chen Y, Huang ST, Hsu TC, Peng LN, Hsiao FY, Chen LK. Detecting Suspected Prescribing Cascades by Prescription Sequence Symmetry Analysis of Nationwide Real-World Data. *J Am Med Dir Assoc*. Mar 2022;23(3):468-474 e6. doi:10.1016/j.jamda.2021.06.035

10. Kalisch LM. The prescribing cascade. *Australian Prescriber*. 2011;(34):162-6.

11. Hallas J. Evidence of depression provoked by cardiovascular medication: a prescription sequence symmetry analysis. *Epidemiology*. Sep 1996;7(5):478-84.

12. Thiessen BQ, Wallace SM, Blackburn JL, Wilson TW, Bergman U. Increased prescribing of antidepressants subsequent to beta-blocker therapy. *Arch Intern Med*. Nov 1990;150(11):2286-90.

13. Johnell K, Fastbom J. The association between use of cardiovascular drugs and antidepressants: a nationwide register-based study. *Eur J Clin Pharmacol*. Nov 2008;64(11):1119-24. doi:10.1007/s00228-008-0541-3

14. Rasmussen L, Hallas J, Madsen KG, Pottegard A. Cardiovascular drugs and erectile dysfunction - a symmetry analysis. *Br J Clin Pharmacol*. Nov 2015;80(5):1219-23. doi:10.1111/bcp.12696

15. Savage RD, Visentin JD, Bronskill SE, et al. Evaluation of a Common Prescribing Cascade of Calcium Channel Blockers and Diuretics in Older Adults With Hypertension. *JAMA Intern Med*. May 1 2020;180(5):643-651. doi:10.1001/jamainternmed.2019.7087

16. Vouri SM, Jiang X, Manini TM, et al. Magnitude of and Characteristics Associated With the Treatment of Calcium Channel Blocker-Induced Lower-Extremity Edema With Loop Diuretics. *JAMA Netw Open*. Dec 2 2019;2(12):e1918425. doi:10.1001/jamanetworkopen.2019.18425

17. Vouri SM, Morris EJ, Usmani SA, et al. Evaluation of the key prescription sequence symmetry analysis assumption using the calcium channel blocker: Loop diuretic prescribing cascade. *Pharmacoepidemiol Drug Saf*. Jan 2022;31(1):72-81. doi:10.1002/pds.5362

18. van Dijk KN, de Vries CS, van den Berg PB, Dijkema AM, Brouwers JR, de Jong-van den Berg LT. Constipation as an adverse effect of drug use in nursing home patients: an overestimated risk. *Br J Clin Pharmacol*. Sep 1998;46(3):255-61. doi:10.1046/j.1365-2125.1998.00777.x

19. Gurwitz JH, Kalish SC, Bohn RL, et al. Thiazide diuretics and the initiation of anti-gout therapy. *J Clin Epidemiol*. Aug 1997;50(8):953-9. doi:10.1016/s0895-4356(97)00101-7

20. Nishtala PS, Chyou TY. Exploring New Zealand prescription data using sequence symmetry analyses for predicting adverse drug reactions. *J Clin Pharm Ther*. Apr 2017;42(2):189-194. doi:10.1111/jcpt.12491

21. Kwan D, Farrell B. Polypharmacy: optimizing medication use in elderly patients. *CGS Journal of CME*. 2014;4(1):21-7.

22. Kalisch Ellett LM, Pratt NL, Barratt JD, Rowett D, Roughead EE. Risk of medication-associated initiation of oxybutynin in elderly men and women. *J Am Geriatr Soc*. Apr 2014;62(4):690-5. doi:10.1111/jgs.12741

23. Silwer L, Petzold M, Hallas J, Lundborg CS. Statins and nonsteroidal anti-inflammatory drugs-an analysis of prescription symmetry. *Pharmacoepidemiol Drug Saf*. Jul 2006;15(7):510-1. doi:10.1002/pds.1250

24. Garrison SR, Dormuth CR, Morrow RL, Carney GA, Khan KM. Nocturnal leg cramps and prescription use that precedes them: a sequence symmetry analysis. *Arch Intern Med*. Jan 23 2012;172(2):120-6. doi:10.1001/archinternmed.2011.1029

25. Khayznikov M, Hemachrandra K, Pandit R, Kumar A, Wang P, Glueck CJ. Statin Intolerance Because of Myalgia, Myositis, Myopathy, or Myonecrosis Can in Most Cases be Safely Resolved by Vitamin D Supplementation. *N Am J Med Sci*. Mar 2015;7(3):86-93. doi:10.4103/1947-2714.153919

26. Takada M, Fujimoto M, Yamazaki K, Takamoto M, Hosomi K. Association of statin use with sleep disturbances: data mining of a spontaneous reporting database and a prescription database. *Drug Saf*. Jun 2014;37(6):421-31. doi:10.1007/s40264-014-0163-x

27. Fujimoto M, Higuchi T, Hosomi K, Takada M. Association of statin use with storage lower urinary tract symptoms (LUTS): data mining of prescription database. *Int J Clin Pharmacol Ther*. Sep 2014;52(9):762-9. doi:10.5414/CP202113

28. Lindberg G, Hallas J. Cholesterol-lowering drugs and antidepressants--a study of prescription symmetry. *Pharmacoepidemiol Drug Saf*. Nov 1998;7(6):399-402. doi:10.1002/(SICI)1099-1557(199811/12)7:6<399::AID-PDS385>3.0.CO;2-C

29. Ko HHT, Lareu RR, Dix BR, Hughes JD, Parsons RW. A sequence symmetry analysis of the interrelationships between statins, diabetes and skin infections. *Br J Clin Pharmacol*. Nov 2019;85(11):2559-2567. doi:10.1111/bcp.14077

30. Pratt N, Chan EW, Choi NK, et al. Prescription sequence symmetry analysis: assessing risk, temporality, and consistency for adverse drug reactions across datasets in five countries. *Pharmacoepidemiol Drug Saf*. Aug 2015;24(8):858-64. doi:10.1002/pds.3780

31. Yokoyama S, Tanaka Y, Hosomi K, Takada M. Polypharmacy Is Associated With Amiodarone-Induced Hypothyroidism. *Int J Med Sci*. 2021;18(15):3574-3580. doi:10.7150/ijms.61412

32. Ponte ML, Wachs L, Wachs A, Serra HA. Prescribing cascade. A proposed new way to evaluate it. *Medicina (B Aires)*. 2017;77(1):13-16. Prescripcion en cascada. Una nueva propuesta para evaluarla.

33. Bytzer P, Hallas J. Drug-induced symptoms of functional dyspepsia and nausea. A symmetry analysis of one million prescriptions. *Aliment Pharmacol Ther*. Nov 2000;14(11):1479-84. doi:10.1046/j.1365-2036.2000.00862.x

34. Petri H, de Vet HC, Naus J, Urquhart J. Prescription sequence analysis: a new and fast method for assessing certain adverse reactions of prescription drugs in large populations. *Stat Med*. Nov 1988;7(11):1171-5. doi:10.1002/sim.4780071110

35. Jan M, Brothers E, Nakagawa TA. Midodrine overdose in children: a case report and review of treatment for hypertensive emergencies. *Transl Pediatr*. Sep 2021;10(9):2398-2401. doi:10.21037/tp-21-153

36. Tsiropoulos I, Andersen M, Hallas J. Adverse events with use of antiepileptic drugs: a prescription and event symmetry analysis. *Pharmacoepidemiol Drug Saf*. Jun 2009;18(6):483-91. doi:10.1002/pds.1736

37. Gau CS, Chang CJ, Tsai FJ, Chao PF, Gau SS. Association between mood stabilizers and hypothyroidism in patients with bipolar disorders: a nested, matched case-control study. *Bipolar Disord*. May 2010;12(3):253-63. doi:10.1111/j.1399-5618.2010.00814.x

38. Lai EC, Yang YH, Lin SJ, Hsieh CY. Use of antiepileptic drugs and risk of hypothyroidism. *Pharmacoepidemiol Drug Saf*. Oct 2013;22(10):1071-9. doi:10.1002/pds.3498

39. Read SH, Giannakeas V, Pop P, et al. Evidence of a gabapentinoid and diuretic prescribing cascade among older adults with lower back pain. *J Am Geriatr Soc*. Oct 2021;69(10):2842-2850. doi:10.1111/jgs.17312

40. Holloman LC, Marder SR. Management of acute extrapyramidal effects induced by antipsychotic drugs. *Am J Health Syst Pharm*. Nov 1 1997;54(21):2461-77. doi:10.1093/ajhp/54.21.2461

41. Hirano Y. Risk of Extrapyramidal Syndromes Associated With Psychotropic Polypharmacy: A Study Based on Large-Scale Japanese Claims Data. *Ther Innov Regul Sci*. Mar 2020;54(2):259-268. doi:10.1007/s43441-019-00026-0

42. Avorn J, Bohn RL, Mogun H, et al. Neuroleptic drug exposure and treatment of parkinsonism in the elderly: a case-control study. *Am J Med*. Jul 1995;99(1):48-54. doi:10.1016/s0002-9343(99)80104-1

43. Rochon PA, Stukel TA, Sykora K, et al. Atypical antipsychotics and parkinsonism. *Arch Intern Med*. Sep 12 2005;165(16):1882-8. doi:10.1001/archinte.165.16.1882

44. Lai EC, Hsieh CY, Kao Yang YH, Lin SJ. Detecting potential adverse reactions of sulpiride in schizophrenic patients by prescription sequence symmetry analysis. *PLoS One*. 2014;9(2):e89795. doi:10.1371/journal.pone.0089795

45. Kalisch Ellett LM, Pratt NL, Kerr M, Roughead EE. Antipsychotic polypharmacy in older Australians. *Int Psychogeriatr*. Apr 2018;30(4):539-546. doi:10.1017/S1041610217001934

46. Iaboni A, Fitzgerald P, Rodin G. Special Issues in Psychopharmacology: The Elderly. *Psychopharmacology in Oncology and Palliative Care: A Practical Manual*. 2014.

47. Pratt N, Andersen M, Bergman U, et al. Multi-country rapid adverse drug event assessment: the Asian Pharmacoepidemiology Network (AsPEN) antipsychotic and acute hyperglycaemia study. *Pharmacoepidemiol Drug Saf*. Sep 2013;22(9):915-24. doi:10.1002/pds.3440

48. Man KKC, Shao SC, Chaiyakunapruk N, et al. Metabolic events associated with the use of antipsychotics in children, adolescents and young adults: a multinational sequence symmetry study. *Eur Child Adolesc Psychiatry*. Jan 2022;31(1):99-120. doi:10.1007/s00787-020-01674-6

49. Takeuchi Y, Kajiyama K, Ishiguro C, Uyama Y. Atypical Antipsychotics and the Risk of Hyperlipidemia: A Sequence Symmetry Analysis. *Drug Saf*. Jul 2015;38(7):641-50. doi:10.1007/s40264-015-0298-4

50. Yokoyama S, Wakamoto S, Tanaka Y, Nakagawa C, Hosomi K, Takada M. Association Between Antipsychotics and Osteoporosis Based on Real-World Data. *Ann Pharmacother*. Oct 2020;54(10):988-995. doi:10.1177/1060028020913974

51. Park KR, Kim KB, Baek YH, et al. Signal detection of benzodiazepine use and risk of dementia: sequence symmetry analysis using South Korean national healthcare database. *Int J Clin Pharm*. Dec 2018;40(6):1568-1576. doi:10.1007/s11096-018-0739-0

52. Takada M, Fujimoto M, Hosomi K. Association between Benzodiazepine Use and Dementia: Data Mining of Different Medical Databases. *Int J Med Sci*. 2016;13(11):825-834. doi:10.7150/ijms.16185

53. Onder G, Bonassi S, Abbatecola AM, et al. High prevalence of poor quality drug prescribing in older individuals: a nationwide report from the Italian Medicines Agency (AIFA). *J Gerontol A Biol Sci Med Sci*. Apr 2014;69(4):430-7. doi:10.1093/gerona/glt118

54. Sultana J, Cutroneo P, Trifiro G. Clinical and economic burden of adverse drug reactions. *J Pharmacol Pharmacother*. Dec 2013;4(Suppl 1):S73-7. doi:10.4103/0976-500X.120957

55. Gill SS, Mamdani M, Naglie G, et al. A prescribing cascade involving cholinesterase inhibitors and anticholinergic drugs. *Arch Intern Med*. Apr 11 2005;165(7):808-13. doi:10.1001/archinte.165.7.808

56. Lampela P, Taipale H, Hartikainen S. Use of Cholinesterase Inhibitors Increases Initiation of Urinary Anticholinergics in Persons with Alzheimer's Disease. *J Am Geriatr Soc*. Jul 2016;64(7):1510-2. doi:10.1111/jgs.14220

57. Venalainen O, Bell JS, Kirkpatrick CM, Nishtala PS, Liew D, Ilomaki J. Adverse Drug Reactions Associated With Cholinesterase Inhibitors-Sequence Symmetry Analyses Using Prescription Claims Data. *J Am Med Dir Assoc*. Feb 1 2017;18(2):186-189. doi:10.1016/j.jamda.2016.11.002

58. Rosenberg J, Rochon PA, Gill SS. Unveiling a prescribing cascade in an older man. *J Am Geriatr Soc*. Mar 2014;62(3):580-1. doi:10.1111/jgs.12714

59. Narayan SW, Pearson SA, Litchfield M, et al. Anticholinergic medicines use among older adults before and after initiating dementia medicines. *Br J Clin Pharmacol*. Sep 2019;85(9):1957-1963. doi:10.1111/bcp.13976

60. Vouri SM, Possinger MC, Usmani S, Solberg LM, Manini T. Evaluation of the Potential Acetylcholinesterase Inhibitor-Induced Rhinorrhea Prescribing Cascade. *J Am Geriatr Soc*. Feb 2020;68(2):440-441. doi:10.1111/jgs.16224

61. Vouri SM, Chung JM, Binder EF. Successful intervention to mitigate an acetylcholinesterase inhibitor-induced rhinorrhea prescribing cascade: a case report. *J Clin Pharm Ther*. Jun 2017;42(3):370-371. doi:10.1111/jcpt.12511

62. Thacker EL, Schneeweiss S. Initiation of acetylcholinesterase inhibitors and complications of chronic airways disorders in elderly patients. *Drug Saf*. 2006;29(11):1077-85. doi:10.2165/00002018-200629110-00007

63. Movig KL, Leufkens HG, Belitser SV, Lenderink AW, Egberts AC. Selective serotonin reuptake inhibitor-induced urinary incontinence. *Pharmacoepidemiol Drug Saf*. Jun 2002;11(4):271-9. doi:10.1002/pds.705

64. Dunvald AD, Henriksen DP, Hallas J, Christensen MMH, Lund LC. Selective serotonin reuptake inhibitors and the risk of restless legs syndrome: a symmetry analysis. *Eur J Clin Pharmacol*. May 2020;76(5):719-722. doi:10.1007/s00228-020-02847-7

65. Pratt N, Roughead E. Assessment of Medication Safety Using Only Dispensing Data. *Curr Epidemiol Rep*. 2018;5(4):357-369. doi:10.1007/s40471-018-0176-6

66. Rababa M, Al-Ghassani AA, Kovach CR, Dyer EM. Proton Pump Inhibitors and the Prescribing Cascade. *J Gerontol Nurs*. Apr 2016;42(4):23-31; quiz 32-3. doi:10.3928/00989134-20151218-04

67. Brandt-Christensen M, Kvist K, Nilsson FM, Andersen PK, Kessing LV. Treatment with antidepressants and lithium is associated with increased risk of treatment with antiparkinson drugs: a pharmacoepidemiological study. *J Neurol Neurosurg Psychiatry*. Jun 2006;77(6):781-3. doi:10.1136/jnnp.2005.083345

68. Marras C, Herrmann N, Fischer HD, et al. Lithium Use in Older Adults is Associated with Increased Prescribing of Parkinson Medications. *Am J Geriatr Psychiatry*. Apr 2016;24(4):301-9. doi:10.1016/j.jagp.2015.11.004

69. Singh G, Magny S, Singh H, Singh S, Virk IS. Differentiating Between Lithium Tremors and Extrapyramidal Tremors in a Patient on Long-Term Antipsychotics and Lithium Medication. *Cureus*. Jul 2023;15(7):e42406. doi:10.7759/cureus.42406

70. Wang Y, Bos JH, Schuiling-Veninga CCM, et al. Neuropsychiatric safety of varenicline in the general and COPD population with and without psychiatric disorders: a retrospective cohort study in a real-world setting. *BMJ Open*. May 25 2021;11(5):e042417. doi:10.1136/bmjopen-2020-042417

71. Wang Y, van Boven JFM, Bos JHJ, et al. Risk of neuropsychiatric adverse events associated with varenicline treatment for smoking cessation among Dutch population: A sequence symmetry analysis. *Pharmacoepidemiol Drug Saf*. Feb 2022;31(2):158-166. doi:10.1002/pds.5351

72. Kivrak Y, Guvenc TS, Akbulut N, et al. Accelerated hypertension after venlafaxine usage. *Case Rep Psychiatry*. 2014;2014:659715. doi:10.1155/2014/659715

73. Thase ME. Effects of venlafaxine on blood pressure: a meta-analysis of original data from 3744 depressed patients. *J Clin Psychiatry*. Oct 1998;59(10):502-8. doi:10.4088/jcp.v59n1002

74. Maura G, Billionnet C, Coste J, Weill A, Neumann A, Pariente A. Non-bleeding Adverse Events with the Use of Direct Oral Anticoagulants: A Sequence Symmetry Analysis. *Drug Saf*. Sep 2018;41(9):881-897. doi:10.1007/s40264-018-0668-9

75. Yokoyama S, Ieda S, Nagano M, et al. Association between oral anticoagulants and osteoporosis: Real-world data mining using a multi-methodological approach. *Int J Med Sci*. 2020;17(4):471-479. doi:10.7150/ijms.39523

76. Takada M, Fujimoto M, Hosomi K. Difference in risk of gastrointestinal complications between users of enteric-coated and buffered low-dose aspirin. *Int J Clin Pharmacol Ther*. Mar 2014;52(3):181-91. doi:10.5414/CP201997

77. Hachiken H, Murai A, Wada K, Kuwahara T, Hosomi K, Takada M. Difference between the frequencies of antisecretory drug prescriptions in users of buffered vs. enteric-coated low-dose aspirin therapies. *Int J Clin Pharmacol Ther*. Oct 2013;51(10):807-15. doi:10.5414/CP201914

78. Saito T, Ohnuma K, Suzuki H, et al. Polyarthropathy in type 2 diabetes patients treated with DPP4 inhibitors. *Diabetes Res Clin Pract*. Oct 2013;102(1):e8-e12. doi:10.1016/j.diabres.2013.07.010

79. Gadzhanova S, Pratt N, Roughead E. Use of SGLT2 inhibitors for diabetes and risk of infection: Analysis using general practice records from the NPS MedicineWise MedicineInsight program. *Diabetes Res Clin Pract*. Aug 2017;130:180-185. doi:10.1016/j.diabres.2017.06.018

80. Lega IC, Bronskill SE, Campitelli MA, et al. Sodium glucose cotransporter 2 inhibitors and risk of genital mycotic and urinary tract infection: A population-based study of older women and men with diabetes. *Diabetes Obes Metab*. Nov 2019;21(11):2394-2404. doi:10.1111/dom.13820

81. Adimadhyam S, Schumock GT, Calip GS, Smith Marsh DE, Layden BT, Lee TA. Increased risk of mycotic infections associated with sodium-glucose co-transporter 2 inhibitors: a prescription sequence symmetry analysis. *Br J Clin Pharmacol*. Jan 2019;85(1):160-168. doi:10.1111/bcp.13782

82. Lund LC, Hojlund M, Henriksen DP, Hallas J, Kristensen KB. Sodium-glucose cotransporter-2 inhibitors and the risk of gout: A Danish population based cohort study and symmetry analysis. *Pharmacoepidemiol Drug Saf*. Oct 2021;30(10):1391-1395. doi:10.1002/pds.5252

83. Subramaniam K, Joseph MP, Babu LA. A Common Drug Causing a Common Side Effect at an Uncommon Time: Metformin-Induced Chronic Diarrhea and Weight Loss After Years of Treatment. *Clin Diabetes*. Apr 2021;39(2):237-240. doi:10.2337/cd20-0101

84. Kim J, Ahn CW, Fang S, Lee HS, Park JS. Association between metformin dose and vitamin B12 deficiency in patients with type 2 diabetes. *Medicine (Baltimore)*. Nov 2019;98(46):e17918. doi:10.1097/MD.0000000000017918

85. Roughead EE, Chan EW, Choi NK, et al. Variation in Association Between Thiazolidinediones and Heart Failure Across Ethnic Groups: Retrospective analysis of Large Healthcare Claims Databases in Six Countries. *Drug Saf*. Sep 2015;38(9):823-31. doi:10.1007/s40264-015-0318-4

86. I AW, Pratt NL, Kalisch LM, Roughead EE. Comparing time to adverse drug reaction signals in a spontaneous reporting database and a claims database: a case study of rofecoxib-induced myocardial infarction and rosiglitazone-induced heart failure signals in Australia. *Drug Saf*. Jan 2014;37(1):53-64. doi:10.1007/s40264-013-0124-9

87. Roughead EE, Chan EW, Choi NK, et al. Proton pump inhibitors and risk of Clostridium difficile infection: a multi-country study using sequence symmetry analysis. *Expert Opin Drug Saf*. Dec 2016;15(12):1589-1595. doi:10.1080/14740338.2016.1238071

88. Ayuk J, Gittoes NJ. Treatment of hypomagnesemia. *Am J Kidney Dis*. Apr 2014;63(4):691-5. doi:10.1053/j.ajkd.2013.07.025

89. Park SK, Baek YH, Pratt N, Kalisch Ellett L, Shin JY. The Uncertainty of the Association Between Proton Pump Inhibitor Use and the Risk of Dementia: Prescription Sequence Symmetry Analysis Using a Korean Healthcare Database Between 2002 and 2013. *Drug Saf*. Jun 2018;41(6):615-624. doi:10.1007/s40264-018-0638-2

90. Janetzki JL, Sykes MJ, Ward MB, Pratt NL. Proton pump inhibitors may contribute to progression or development of chronic obstructive pulmonary disease-A sequence symmetry analysis approach. *J Clin Pharm Ther*. Dec 2021;46(6):1687-1694. doi:10.1111/jcpt.13520

91. Salazar JA, Poon I, Nair M. Clinical consequences of polypharmacy in elderly: expect the unexpected, think the unthinkable. *Expert Opin Drug Saf*. Nov 2007;6(6):695-704. doi:10.1517/14740338.6.6.695

92. Avorn J, Gurwitz JH, Bohn RL, Mogun H, Monane M, Walker A. Increased incidence of levodopa therapy following metoclopramide use. *JAMA*. Dec 13 1995;274(22):1780-2.

93. King CE, Pratt NL, Craig N, et al. Detecting Medicine Safety Signals Using Prescription Sequence Symmetry Analysis of a National Prescribing Data Set. *Drug Saf*. Aug 2020;43(8):787-795. doi:10.1007/s40264-020-00940-5

94. Wahab IA, Pratt NL, Ellett LK, Roughead EE. Sequence Symmetry Analysis as a Signal Detection Tool for Potential Heart Failure Adverse Events in an Administrative Claims Database. *Drug Saf*. Apr 2016;39(4):347-54. doi:10.1007/s40264-015-0391-8

95. Cole JL. Steroid-Induced Sleep Disturbance and Delirium: A Focused Review for Critically Ill Patients. *Fed Pract*. Jun 2020;37(6):260-267.

96. Huynh G, Reinert JP. Pharmacological Management of Steroid-Induced Psychosis: A Review of Patient Cases. *J Pharm Technol*. Apr 2021;37(2):120-126. doi:10.1177/8755122520978534

97. Parian A, Ha CY. Older age and steroid use are associated with increasing polypharmacy and potential medication interactions among patients with inflammatory bowel disease. *Inflamm Bowel Dis*. Jun 2015;21(6):1392-400. doi:10.1097/MIB.0000000000000391

98. Corrao G, Botteri E, Bagnardi V, et al. Generating signals of drug-adverse effects from prescription databases and application to the risk of arrhythmia associated with antibacterials. *Pharmacoepidemiol Drug Saf*. Jan 2005;14(1):31-40. doi:10.1002/pds.1019

99. Veazie S, Peterson K, Ansari Y, et al. Fludrocortisone for orthostatic hypotension. *Cochrane Database Syst Rev*. May 17 2021;5(5):CD012868. doi:10.1002/14651858.CD012868.pub2

100. Azoulay L, Blais L, Koren G, LeLorier J, Berard A. Isotretinoin and the risk of depression in patients with acne vulgaris: a case-crossover study. *J Clin Psychiatry*. Apr 2008;69(4):526-32. doi:10.4088/jcp.v69n0403

101. Sturkenboom MC, Middelbeek A, de Jong van den Berg LT, van den Berg PB, Stricker BH, Wesseling H. Vulvo-vaginal candidiasis associated with acitretin. *J Clin Epidemiol*. Aug 1995;48(8):991-7. doi:10.1016/0895-4356(94)00239-m

102. Al Odhayani A, Tourkmani A, Alshehri M, Alqahtani H, Mishriky A. Potentially inappropriate medications prescribed for elderly patients through family physicians. *Saudi J Biol Sci*. Jan 2017;24(1):200-207. doi:10.1016/j.sjbs.2016.05.006

103. Lin D, Kramer JR, Ramsey D, et al. Oral bisphosphonates and the risk of Barrett's esophagus: case-control analysis of US veterans. *Am J Gastroenterol*. Oct 2013;108(10):1576-83. doi:10.1038/ajg.2013.222

104. Garcia Rodriguez LA, Jick H. Risk of upper gastrointestinal bleeding and perforation associated with individual non-steroidal anti-inflammatory drugs. *Lancet*. Mar 26 1994;343(8900):769-72. doi:10.1016/s0140-6736(94)91843-0

105. Caughey GE, Roughead EE, Pratt N, Shakib S, Vitry AI, Gilbert AL. Increased risk of hip fracture in the elderly associated with prochlorperazine: is a prescribing cascade contributing? *Pharmacoepidemiol Drug Saf*. Sep 2010;19(9):977-82. doi:10.1002/pds.2009

106. Gurwitz JH, Avorn J, Bohn RL, Glynn RJ, Monane M, Mogun H. Initiation of antihypertensive treatment during nonsteroidal anti-inflammatory drug therapy. *JAMA*. Sep 14 1994;272(10):781-6.

107. Arfe A, Scotti L, Varas-Lorenzo C, et al. Non-steroidal anti-inflammatory drugs and risk of heart failure in four European countries: nested case-control study. *BMJ*. Sep 28 2016;354:i4857. doi:10.1136/bmj.i4857

108. Semenkovich K, Chockalingam R, Scherrer JF, et al. Prescription Opioid Analgesics Increase Risk of Major Depression: New Evidence, Plausible Neurobiological Mechanisms and Management to Achieve Depression Prophylaxis. *Mo Med*. Mar-Apr 2014;111(2):148-154.

109. Henriksen DP, Davidsen JR, Christiansen A, Laursen CB, Damkier P, Hallas J. Inhaled Corticosteroids and Systemic or Topical Antifungal Therapy: A Symmetry Analysis. *Ann Am Thorac Soc*. Jun 2017;14(6):1045-1047. doi:10.1513/AnnalsATS.201612-1043LE

110. Petri H, Kessels F, Kamakura T. Markers of adverse drug reactions in medication histories. An analysis of inhaled steroid utilization. *Pharm Weekbl Sci*. Apr 26 1991;13(2):97-106. doi:10.1007/BF01974988

111. van Boven JF, de Jong-van den Berg LT, Vegter S. Inhaled corticosteroids and the occurrence of oral candidiasis: a prescription sequence symmetry analysis. *Drug Saf*. Apr 2013;36(4):231-6. doi:10.1007/s40264-013-0029-7

112. Winkel JS, Damkier P, Hallas J, Henriksen DP. Treatment with montelukast and antidepressive medication-a symmetry analysis. *Pharmacoepidemiol Drug Saf*. Dec 2018;27(12):1409-1415. doi:10.1002/pds.4638

113. Fox CW, Khaw CL, Gerke AK, Lund BC. Montelukast and neuropsychiatric events - a sequence symmetry analysis. *J Asthma*. Dec 2022;59(12):2360-2366. doi:10.1080/02770903.2021.2018705

114. Sica DA. Alpha1-adrenergic blockers: current usage considerations. *J Clin Hypertens (Greenwich)*. Dec 2005;7(12):757-62. doi:10.1111/j.1524-6175.2005.05300.x

115. Arango Toro O, Nohales Taurines G, Cortadellas Angel R, Castro Santamaria R, Gelabert Mas A. [Management of hyposalivation caused by oxybutynin chloride in the treatment of the unstable bladder]. *Actas Urol Esp*. Feb 1998;22(2):124-30. Manejo de la hiposialia producida por el cloruro de oxibutinina en el tratamiento de la vejiga inestable.

116. Dyson TE, Cantrell MA, Lund BC. Lack of Association between 5alpha-Reductase Inhibitors and Depression. *J Urol*. Oct 2020;204(4):793-798. doi:10.1097/JU.0000000000001079

117. Monane M, Bohn RL, Gurwitz JH, Glynn RJ, Choodnovskiy I, Avorn J. Topical glaucoma medications and cardiovascular risk in the elderly. *Clin Pharmacol Ther*. Jan 1994;55(1):76-83. doi:10.1038/clpt.1994.13
